# Single-cell RNA-sequencing of differentiating iPS cells reveals dynamic genetic effects on gene expression

**DOI:** 10.1038/s41467-020-14457-z

**Published:** 2020-02-10

**Authors:** Anna S. E. Cuomo, Daniel D. Seaton, Davis J. McCarthy, Iker Martinez, Marc Jan Bonder, Jose Garcia-Bernardo, Shradha Amatya, Pedro Madrigal, Abigail Isaacson, Florian Buettner, Andrew Knights, Kedar Nath Natarajan, HipSci Consortium, Chukwuma A. Agu, Alex Alderton, Petr Danecek, Rachel Denton, Richard Durbin, Daniel J. Gaffney, Angela Goncalves, Reena Halai, Sarah Harper, Christopher M. Kirton, Anja Kolb-Kokocinski, Andreas Leha, Shane A. McCarthy, Yasin Memari, Minal Patel, Ewan Birney, Francesco Paolo Casale, Laura Clarke, Peter W. Harrison, Helena Kilpinen, Ian Streeter, Davide Denovi, Ruta Meleckyte, Natalie Moens, Fiona M. Watt, Willem H. Ouwehand, Angus I. Lamond, Dalila Bensaddek, Philip Beales, Ludovic Vallier, John C. Marioni, Mariya Chhatriwala, Oliver Stegle

**Affiliations:** 1 European Molecular Biology Laboratory, European Bioinformatics Institute, Wellcome Genome Campus, CB10 1SD Hinxton, Cambridge, UK; 2 St Vincent’s Institute of Medical Research, Fitzroy, Victoria 3065 Australia; 3 Wellcome Sanger Institute, Wellcome Genome Campus, Hinxton, CB10 1SA UK; 4 Wellcome Trust—MRC Cambridge Stem Cell Institute, Anne McLaren Laboratory, University of Cambridge, Cambridge, CB2 0SZ UK; 5 Department of Surgery, University of Cambridge, Cambridge, CB2 0QQ UK; 6 Cancer Research UK Cambridge Institute, University of Cambridge, Cambridge, UK; 7 Division of Computational Genomics and Systems Genetics, German Cancer Research Center (DKFZ), 69120 Heidelberg, Germany; 8 European Molecular Biology Laboratory, Genome Biology Unit, 69117 Heidelberg, Germany; 9 Present Address: GeneLab, AWG Multi-Omics/System Biology, NASA Ames Research Center, Moffett Field, California, USA; 10 Present Address: Danish Institute of Advanced Study (D-IAS), Functional Genomics and Metabolism Unit, University of Southern Denmark, Odense, Denmark; 11 King’s College London, Strand Ln, London, WC2R 2LS United Kingdom; 12 Department of Haematology, University of Cambridge and National Health Service Blood and Transplant, Hills Rd, Cambridge, CB2 0XY United Kingdom; 13 Centre for Gene Regulation and Expression, School of Life Sciences, University of Dundee, Dow St, Dundee, DD1 5EH United Kingdom; 14 University College London, Gower St, Bloomsbury, London, WC1E 6BT United Kingdom

**Keywords:** Functional genomics, Genetic association study, Quantitative trait loci, Induced pluripotent stem cells

## Abstract

Recent developments in stem cell biology have enabled the study of cell fate decisions in early human development that are impossible to study in vivo. However, understanding how development varies across individuals and, in particular, the influence of common genetic variants during this process has not been characterised. Here, we exploit human iPS cell lines from 125 donors, a pooled experimental design, and single-cell RNA-sequencing to study population variation of endoderm differentiation. We identify molecular markers that are predictive of differentiation efficiency of individual lines, and utilise heterogeneity in the genetic background across individuals to map hundreds of expression quantitative trait loci that influence expression dynamically during differentiation and across cellular contexts.

## Abstract (web summary)

Studying the genetic effects on early stages of human development is challenging due to a scarcity of biological material. Here, the authors utilise induced pluripotent stem cells from 125 donors to track gene expression changes and expression quantitative trait loci at single cell resolution during in vitro endoderm differentiation.

## Introduction

The early stages of human embryogenesis involve dramatic and dynamic changes in cellular states. However, the extent to which an embryo’s genetic background influences this process has only been determined in a small number of special cases linked to rare large-effect variants that cause developmental disorders. This lack of information is critical—it can provide a deep understanding of how genetic heterogeneity is tolerated in normal development, when controlling the expression of key genes is vital. Additionally, with cellular reprogramming becoming an increasingly used tool in molecular medicine, understanding how inter-individual variability effects such differentiations is key.

Critically, recent technological developments have begun to facilitate such studies in vitro. In particular, the generation of population-scale collections of human induced pluripotent stem cells (iPSCs)^1,2^ has allowed for assessing regulatory genetic variants in pluripotent^1,2^ as well as in differentiated cells^3–5^. In addition, the rapid developments in single-cell RNA-seq now allow for assessing the molecular impact of genetic variability in a continuous manner across early human development.

Here, we use a pooled cell differentiation assay to study endoderm differentiation across a set of human iPSC lines from 125 donor, profiling changes in gene expression via single-cell RNA-sequencing at four developmental timepoints^6^. Our study allows discovery of hundreds of expression Quantitative Trait Loci (eQTL) that vary across differentiation. We generalise approaches from studies of the interaction between genotype and environment (GxE) by leveraging the single-cell resolution of our study to investigate the interplay between genetic factors and cellular states. Finally, we also identify gene expression markers of the differentiation capacity of iPSC lines, highlighting loss of X chromosome inactivation in female cell lines as an important cellular phenotype in this system.

## Results

### Population-scale profiling of differentiating iPS cells

**Fig. 1:**
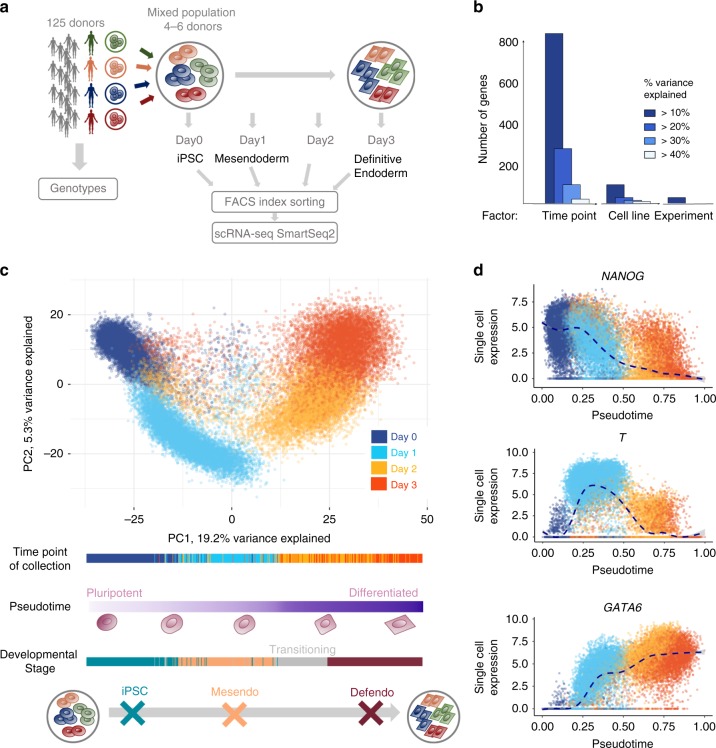
Single-cell endoderm differentiation of pooled iPSC lines. **a** Overview of the experimental design. iPSC lines from 125 genotyped donors were pooled in sets of 4-6, across 28 experiments, followed by differentiation towards definitive endoderm. Cells were sampled every 24 h (Methods) and molecularly profiled using scRNA-seq and FACS. https://github.com/ebiwd/EBI-Icon-fonts by EBI Web Development is licensed under CC BY 4.0. **b** Variance component analysis of 4,546 highly variable genes, using a linear mixed model fit to individual genes to decompose expression variation into time point of collection, cell line and experimental batch (Methods). **c** Top: Principal component analysis of gene expression profiles for 36,044 QC-passing cells. Cells are coloured by the time point of collection. Bottom: Cells are ordered by pseudotime, defined as the first principal component (PC1). From left to right, cells transition from a pluripotent state to definitive endoderm. **d** Single cell expression (*y*-axis) of selected markers for each developmental stage, spanning iPSC (*NANOG*), mesendo (*T*), and defendo (*GATA6*) stages, plotted along pseudotime (*x*-axis).

We considered a panel of well-characterised human iPSC lines derived from 125 unrelated British donors from the Human Induced Pluripotent Stem Cell initiative (HipSci) collection^1^. In order to increase throughput and mitigate the effects of batch variation, we exploited a pooled differentiation assay, combining sets of four to six lines in one well prior to differentiation (28 differentiation experiments performed in total; hereon “experiments”; Fig. 1a, Supplementary Fig. 1, 2). Cells were collected at four differentiation time points (iPSC; one, two and three days post initiation—hereon day0, day1, day2 and day3) and their transcriptomes were assayed using full-length RNA-sequencing (Smart-Seq2^7^) alongside the expression of selected cell surface markers using FACS (TRA-1-60, CXCR4; Supplementary Fig. 3, 4; Methods). Following quality control (QC), 36,044 cells were retained for downstream analysis, across which 11,231 genes were expressed (Supplementary Fig. 5; Methods). Exploiting that each cell line’s genotype acts as a unique barcode, we demultiplexed the pooled cell populations, enabling identification of the cell line of origin for each cell (similar to^8,9^; Methods). At each time point, cells from between 104 and 112 donors were captured, with each donor being represented by an average of 286 cells (after QC, Supplementary Fig. 2**;** Supplementary Data 1, 2; Methods). The success of the differentiation protocol was validated using canonical cell-surface marker expression: consistent with previous studies^10^, an average of 72% cells were TRA-1-60(+) in the undifferentiated state (day0) and an average of 49% of cells were CXCR4(+) three days post differentiation (day3; Supplementary Fig. 3).

Variance component analysis across all genes (using a linear mixed model; Methods) revealed the time point of collection as the main source of variation, followed by the cell line of origin and the experimental batch (Fig. 1b). Consistent with this, the first Principal Component (PC) was strongly associated with differentiation time (Fig. 1c, Supplementary Fig. 6; Methods), motivating its use to order cells by their differentiation status (hereafter “pseudotime”, Fig. 1c). Alternative pseudotime inference methods yielded similar orderings (Supplementary Fig. 7; Methods).

Critically, the expected temporal expression dynamics of marker genes that characterise endoderm differentiation was captured by the ordering of cells along the inferred pseudotime (Fig. 1d). Exploiting these markers of differentiation progress and pseudotime, we assigned 28,971 cells (~80%) to one of three canonical stages of endoderm differentiation: iPSC, mesendoderm (mesendo) and definitive endoderm (defendo) (Fig. 1c, Supplementary Fig. 8; Methods). A smaller fraction of cells (*N* = 7073) could not be confidently assigned to a canonical stage of differentiation; these cells were heavily enriched for those collected at day2, when rapid changes in molecular profiles are expected, reflecting a transitional population of cells.

### Pseudo-temporal ordering yields stage-specific eQTL

**Fig. 2:**
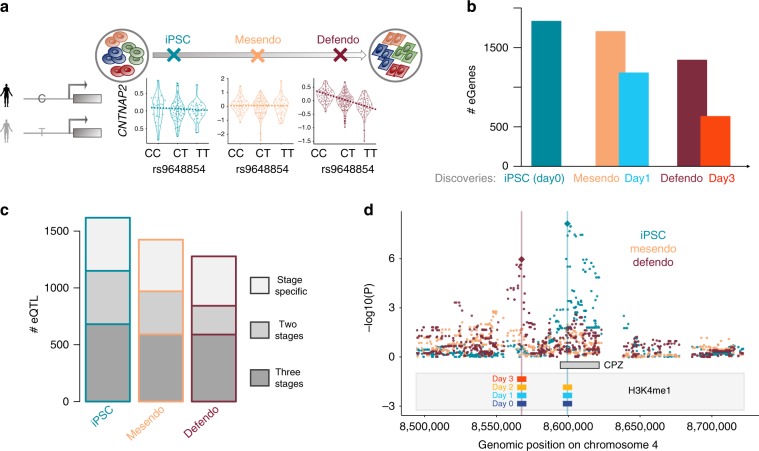
Mapping single-cell eQTL in each developmental stage. **a** Illustration of the single cell eQTL mapping strategy at different stages of differentiation. Shown is an example of an eQTL that is specific to the definitive endoderm (defendo) stage. Boxplots of gene expression stratified by the allelic state of *rs9648854* at each stage, showing an association between *rs9648854* and *CNTNAP2* expression at the defendo stage, but not at earlier stages. https://github.com/ebiwd/EBI-Icon-fonts by EBI Web Development is licensed under CC BY 4.0. **b** Comparison of eQTL mapping using different strata of all cells. Stage definition based on pseudotime ordering increases the number of detectable eQTL, compared to using the corresponding time point of collection. Bars represent number of eGenes (genes with at least one eQTL, at FDR < 10%). **c** Proportion of eQTL that are specific to a single stage, shared across two stages, or observed across all stages (sharing defined as a lead eQTL variant at one stage with nominal significant effects *P* < 0.05 and consistent direction at another stage). **d** A lead switching event consistent with epigenetic remodelling. The overlap of H3K4me1 with the eQTL SNPs across differentiation time points is indicated by the coloured bars.

Motivated by the observation that a substantial fraction of variability in gene expression was explained by cell-line effects (Fig. 1b), we tested for associations between common genetic variants and gene expression at the three defined stages of cell differentiation (Figs. 1c, 2a). Briefly, for each donor, experiment, and differentiation stage, we quantified each gene’s average expression level (Methods), before using a linear mixed model to test for *cis* eQTL, adapting approaches used for bulk RNA-seq profiles (+/− 250 kb, MAF >5%^1^; Methods). In the iPSC population (day0), this identified 1,833 genes with at least one eQTL (denoted eGenes; FDR <10%; 10,840 genes tested; Supplementary Data 3). To validate our approach, we also performed eQTL mapping using deep bulk RNA-sequencing profiles from the same set of iPSC lines (“iPSC bulk”; 10,736 genes tested) generated as part of the HipSci project^1^, yielding consistent eQTL (~70% replication of lead eQTL effects; nominal *P* < 0.05; Methods; Supplementary Data 4). These iPSC eQTL were further confirmed by analysis of scRNA-seq data generated from a subset of 5 experiments using a droplet-based approach (Methods; Supplementary Fig. 9, 10).

Analogously, we mapped eQTL in the mesendo and defendo populations, yielding 1702 and 1342 eGenes, respectively. For comparison, we also performed eQTL mapping in cells collected on day1 and day3—the experimental time points commonly used to identify cells at mesendo and defendo stages^6^. Interestingly, this approach identified markedly fewer eGenes (1181 eGenes at day1, and 631 eGenes at day3), demonstrating the power of using the single-cell RNA-seq profiles to define relatively homogeneous differentiation stages in a data-driven manner (Fig. 2b; Methods; Supplementary Table 1). Notably, this observation did not merely reflect differences in the number of cells or donors considered (Supplementary Fig. 11).

Profiling multiple stages of endoderm differentiation allowed us to assess at which stage along this process individual eQTL can be detected. We observed substantial regulatory and transcriptional remodelling upon iPS differentiation to definitive endoderm, with over 30% of eQTL being specific to a single stage (Fig. 2a, c; Methods), where we considered the pairwise replication of eQTL to define stage-specific effects (nominal *P* < 0.05 and consistent effect direction; Methods). Importantly, we note that stage-specificity of eQTL was not significantly explained by stage-specific gene expression (Supplementary Fig. 12). Our differentiation time course covers developmental stages that have never before been accessible to genetic analyses of molecular traits. Consistent with this, 349 of our eQTL variants at the mesendo and defendo stages have not been reported in either a recent iPSC eQTL study based on bulk RNA-seq^11^, or in a compendium of eQTL identified from 49 tissues as part of the GTEx project^12^ (linkage disequilibrium with lead variants in GTEx, LD: r^2 < ^0.2; Methods; Supplementary Data 3).

In addition to these eQTL, we identified lead switching events for 155 eGenes. Those are two distinct variants for the same gene that are identified as lead eQTL at different stages of differentiation (at LD: r^2 < ^0.2; for example iPSC and defendo in Fig. 2d; Methods). To investigate the potential regulatory role of such variants, we examined whether the corresponding genetic loci also featured changes in histone modifications during differentiation. Specifically, we used ChIP-Sequencing to profile five histone modifications associated with promoter and enhancer usage (H3K27ac, H3K4me1, H3K4me3, H3K27me3, and H3K36me3) in hESCs that were differentiated towards endoderm (using the same protocol employed above) and measured at equivalent time points (i.e. day0, day1, day2, day3; Methods). Intriguingly, for 20 of the lead switching events, we observed corresponding changes in the epigenetic landscape (stage-specific lead variants overlap with stage-specific changes in histone modification status), suggesting a direct mode of action (Fig. 2d).

### Discovery of dynamic eQTL across iPSC differentiation

**Fig. 3:**
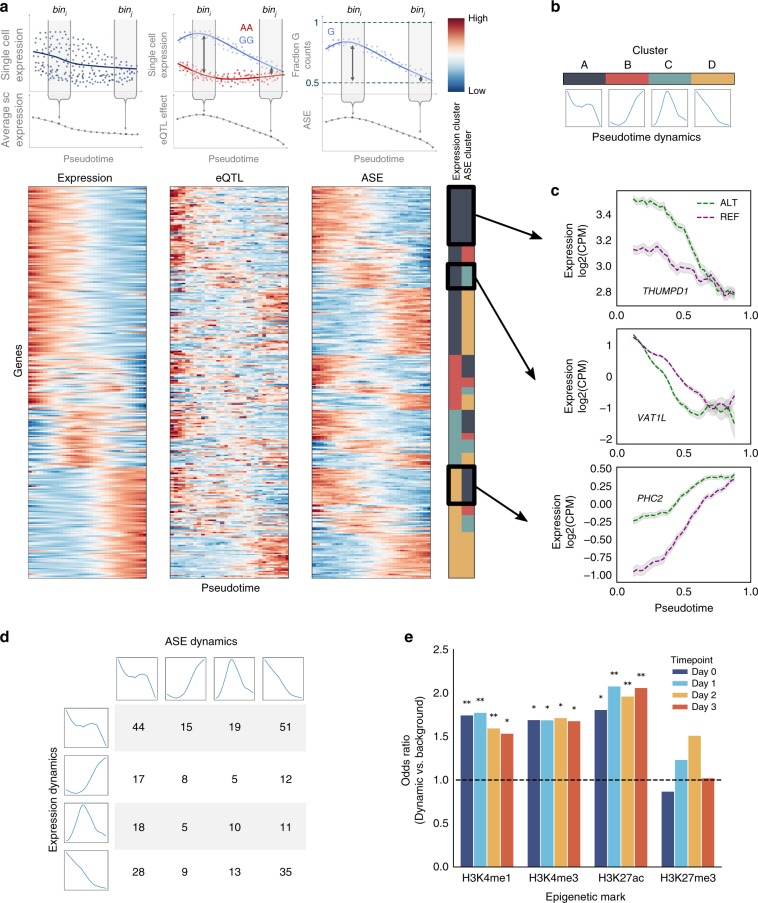
eQTL dynamics during differentiation. **a** Combined analysis of the gene expression, ASE, and eQTL dynamics across pseudotime. Upper panels: Schematic of sliding window approach. Cells are binned according to pseudotime groups, to quantify average expression, perform an eQTL analysis, and quantify average ASE (each bin includes 25% of cells, binned at increments of 2.5%). Lower panels: clustered heatmap of expression levels, eQTL effects, and ASE across pseudotime for the top 311 genes with the strongest dynamic QTL effects (FDR < 1%; out of 785 at FDR < 10%; Methods). For each gene, the expression and the ASE dynamics were jointly grouped using clustering analysis, with 4 clusters. The membership of gene expression and ASE dynamics of these 4 clusters is indicated by colours in the right-hand panel. Values in all heatmaps are z-score normalised by row. For ASE, average ASE values are plotted such that red indicates highest deviation from 0.5. **b** Summary of the identified cluster dynamics, displaying the average dynamic profile of each cluster, computed as the average across z-score normalised gene expression/ASE profiles. **c** Exemplars of the dynamic gene expression and dynamic genetic effects clusters shown in **a**. Shaded regions indicate standard error (+/− 1 SEM; Methods). **d** Number of genes categorised by the combination of expression and ASE cluster from **a**. Average dynamics of expression clusters (rows) and ASE clusters (columns) as in **b** are shown. **e** Overlap of dynamic eQTL variants from **a** with histone marks. The odds ratio compared to the background of all other eQTL variants is shown (*P < 0.01; **P < 1 × 10^−4^; Fisher’s exact test).

The availability of large numbers of cells per donor across the differentiation trajectory enabled the analysis of dynamic changes of eQTL strength at fine-grained resolution. Using a sliding-window approach (25% cells in each window, sliding along pseudotime by a step of 2.5% cells), we explored how the joint set of 4422 eQTL lead variants (4470 SNP-gene pairs) discovered at the iPSC, mesendo, and defendo stages were modulated by developmental time. To do this, we reassessed each eQTL in each window, taking advantage of the full length transcript sequencing to measure allele-specific expression (ASE) in each window (Methods). Here, in each window, we quantified the deviation from 0.5 of the expression of the minor allele at the eQTL (ratio of reads phased to eQTL variants, Methods). Notably, ASE can be quantified in each cell and is independent of expression level, thus mitigating technical correlations between differentiation stage and genetic effect estimates. As a complementary approach, we also considered average expression quantifications per window to estimate genetic effects using eQTL mapping (Methods). Both methods result in a measure of the varying strength of genetic effects along development, or genetic effect dynamics. Reassuringly, the two approaches were highly consistent across pseudotime (Fig. 3a, Supplementary Fig. 13).

To formally test for eQTL effects that change dynamically across differentiation (dynamic QTL), we tested for associations between pseudotime and the genetic effect size using a linear model (genetic effect defined based on ASE at the level of single cells; likelihood ratio test, considering linear and quadratic pseudotime), uncovering a total of 899 time dynamic eQTL (out of the joint set of 4422 eQTL across all stages; FDR < 10%; Methods), including a substantial fraction of eQTL that were not stage-specific (Supplementary Data 3). This complements our earlier analysis based on discrete differentiation stages, which identified substantial stage-specific effects (Fig. 2a,c), by identifying subtle changes in the relationship between genotype and phenotype during differentiation. To further explore this set of genes, we clustered eQTL jointly based on the relative gene expression dynamics (global expression changes along pseudotime, quantified in sliding windows as above, Methods), and on the genetic effect dynamics (Fig. 3a; Methods). This identified four basic dynamic patterns (Fig. 3b): decreasing early (cluster A), decreasing late (cluster B), transiently increasing (cluster C), and increasing (cluster D). As expected, stage-specific eQTL were grouped together in particular clusters (e.g. defendo specific eQTL in cluster D; Supplementary Fig. 14). Notably, the gene expression dynamics and the eQTL dynamics tended to be distinct, demonstrating that gene expression level is not the primary mechanism governing variation in genetic effects. In particular, genetic effects were not most pronounced when gene expression was high (Fig. 3c, d, Supplementary Fig. 15).

Distinct combinations of expression and eQTL dynamics result in different patterns of allelic expression. This is illustrated by the mesendoderm-specific eQTL for *VAT1L*. Overall expression of *VAT1L* decreases during differentiation, but expression of the alternative allele is repressed more quickly than that of the reference allele (Fig. 3c). This illustrates how *cis* regulatory sequence variation can modulates the timing of expression changes in response to differentiation, similar to observations previously made in *C. elegans* using recombinant inbred lines^13^. In other cases, the genetic effect coincides with high or low expression, for example in the cases of *THUMPD1* and *PHC2* (Fig. 3c). These examples illustrate how genetic variation is intimately linked to the dynamics of gene regulation.

We next asked whether dynamic eQTL were located in specific regulatory regions. To do this, we evaluated the overlap of the epigenetic marks defined using the hESC differentiation time series with the dynamic eQTL (Fig. 3e, Supplementary Fig. 16). This revealed an enrichment of dynamic eQTL in H3K27ac, H3K4me1 (i.e., enhancer marks), and H3K4me3 (i.e. promoter) marks compared to non-dynamic eQTL (i.e. eQTL that we identified but did not display dynamic changes along pseudotime, Fig. 3e), consistent with these SNPs being located in active regulatory elements.

### Cellular environment modulates genetic effects on expression

Whilst differentiation was the main source of variation in the dataset, single cell RNA-seq profiles can be used to characterise cell-to-cell variation across a much wider range of cell state dimensions^14–16^. We identified sets of genes that varied in a co-regulated manner using clustering (affinity propagation; 8000 most highly expressed genes; Supplementary Data 5; Methods), which identified 60 modules of co-expressed genes. The resulting modules were enriched for key biological processes such as cell differentiation, cell cycle state (G1/S and G2/M transitions), respiratory metabolism, and sterol biosynthesis (as defined by Gene Ontology annotations; Supplementary Data 6). These functional annotations were further supported by transcription factor binding (e.g., enrichment of SMAD3 and E2F7 targets in the differentiation and cell cycle modules, respectively (Supplementary Table 2, Supplementary Data 7)). Additionally, expression of the cell differentiation module (cluster 6; Supplementary Table 2) was correlated with pseudotime, as expected (R = 0.62; Supplementary Fig. 7C).

**Fig. 4:**
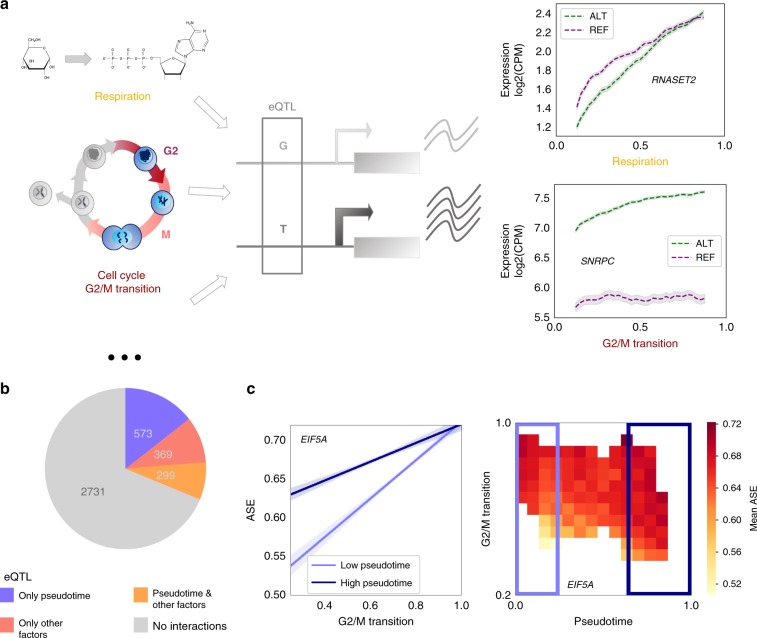
Allele-specific expression reveals interactions with fundamental cellular processes. **a** Illustration of eQTL affected by cellular context. Left: Schematic of cellular contexts affecting a regulatory element containing an eQTL SNP, and thus affecting allele-specific expression. Right: Allele-specific expression variation for two exemplar eQTL SNPs that tag cancer GWAS variants and display GxE interactions (FDR < 10%). The eQTL for *RNASET2* (*rs2247315*) tags a risk variant for basal cell carcinoma, and is responsive to cellular respiration, while that for *SNRPC* (*rs9380455*) tags a risk variant for prostate cancer and is responsive to the cell cycle G2/M transition (Table S13). Cellular contexts for each cell were inferred by GO annotations of coexpression modules (Methods). Shaded regions indicate standard error (+/− 1 SEM; Methods). **b** Results summary: numbers of eQTL (from Fig. 2; Methods) identified as displaying GxE interactions with pseudotime (purple), displaying GxE interactions with other cellular contexts but not with pseudotime, (after appropriately accounting for pseudotime, red), displaying GxE interactions with both pseudotime and at least one other cellular context (yellow), and displaying no GxE interactions at all (grey). Significance is assessed at FDR < 10%. **c** Higher order interaction example: an eQTL variant for *EIF5A* (*rs7503161*) is affected by a GxExE higher order interaction with both pseudotime and the G1/S transition. Left panel: Effects of respiration state on ASE for cells with low and high pseudotime. Lines shown are linear regressions with 95% confidence intervals for the 30% of cells with lowest and highest values for pseudotime. Right panel: Heatmap of averaged ASE for cells falling within the specified windows of pseudotime and respiration state. Only values for windows containing *n* > 30 cells are shown (*n* = 6423 cells in total).

Using the same ASE-based interaction test as applied to identify dynamic QTL, reflecting ASE variation across pseudotime (Fig. 3**;** Methods), we assessed how the genetic regulation of gene expression responded to these cellular contexts. Briefly, we tested for genotype by environment (GxE) interactions using a subset of four co-expression modules as markers of cellular state, while accounting for effects that can be explained by interactions with pseudotime (Fig. 4a; Methods). These four co-expression modules were annotated based on GO term enrichment, and their normalised mean expression levels in each cell were taken as quantitative measures of cell cycle state (G1/S and G2/M transitions) and metabolic pathway activity (respiratory metabolism and sterol biosynthesis; Methods). This approach extends previous work using ASE to discover GxE interactions^17,18^, taking advantage of the resolution provided by single-cell data. We identified 668 eQTL that had an interaction effect with at least one factor (Fig. 4b; FDR < 10%), with many of these eQTL having no evidence for an interaction with differentiation. Indeed, 369 genes had no association with pseudotime, but responded to at least one other factor. Conversely, of the 872 dynamic eQTL, 299 were also associated with GxE effects with other factors, whereas 573 were exclusively associated with pseudotime (Fig. 4b, Supplementary Fig. 17**;** Supplementary Data 8-10; Methods).

These interactions encompass regulatory effects on genes and SNPs with important functional roles. Specifically, 95 interaction eQTL variants overlap with variants previously identified in genome-wide association studies (GWAS, LD *r*^2 > ^0.8; Methods; Supplementary Data 11). For example, an eQTL for *RNASET2* shows sensitivity to cellular respiratory metabolic state (Fig. 4a). This eQTL SNP is in LD (*r*^2^ = 0.86) with a GWAS risk variant for basal cell carcinoma^19^. Furthermore, an eQTL for *SNRPC* showed sensitivity to the G2/M state, and is in LD (*r*^2^ = 0.92) with a GWAS risk variant for prostate cancer^20^ (Fig. 4a). These cellular factors vary not only across cells in the experiments considered here, but also across cells in vivo, across individuals, and across environments. Thus, these examples illustrate the versatility of our single cell dataset and how it can provide regulatory information about variants in contexts beyond early human development.

Finally, we explored whether we could detect higher order interaction effects, where the genetic effect varies with a cellular state in different ways along differentiation, effectively testing for GxExE interactions. To this end, we fitted a linear model with fixed effects for differentiation and each of the factors, plus a combined term (factor x pseudotime, Fig. 4b, c; Methods). This identified 176 genes with significant higher order interactions between a genetic variant, differentiation, and at least one other factor (Fi. 4b, c, Supplementary Fig. 17**;** Supplementary Data 10). One example is an eQTL for *EIF5A*, whose ASE was responsive to G2/M state, especially early in differentiation (Fig. 4c). These results highlight the context-specificity of eQTL, and the power of scRNA-seq in dissecting this specificity within one set of experiments.

### Early markers are predictive of differentiation efficiency

**Fig. 5:**
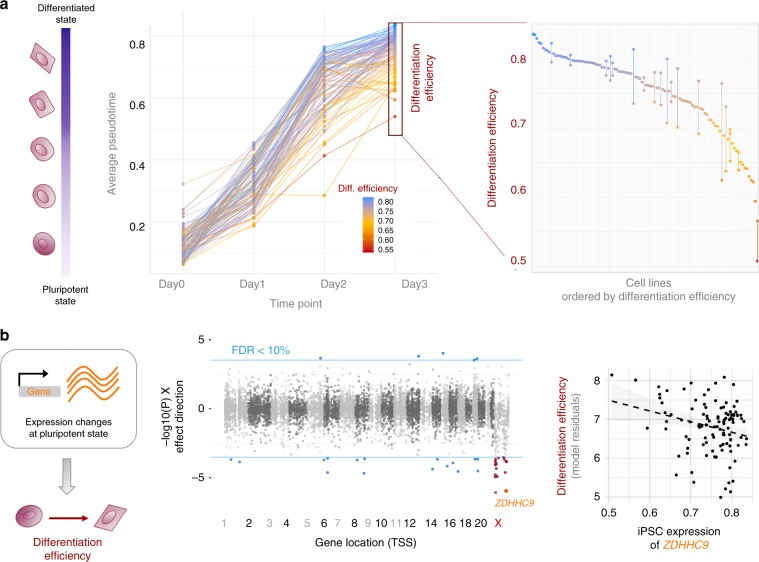
Identification of molecular markers for differentiation efficiency. **a** Variation in differentiation efficiency across cell lines. Left: Differentiation progress over time, showing trajectories for 98 cell lines, coloured by differentiation efficiency. Shown are 98 cell lines with sufficient data at all time points (out of 126, more than 10 cells). Differentiation efficiency of a cell line was defined as the average pseudotime across all cells on day 3. Right: Differentiation efficiency across cell lines (points), and consistency of individual cell lines differentiated in multiple experiments (vertical bars). **b** Associations between iPSC gene expression levels and differentiation efficiency. Left: schematic. Centre: Genome-wide analysis to identify markers of differentiation efficiency, considering iPSC gene expression levels. Displayed are negative log *P*-values signed by the direction of the effect. Horizontal blue lines denote FDR = 10% (Benjamini-Hochberg adjusted). Autosomal genes with significant associations are shown in blue; X chromosome genes with significant associations are shown in red. Right: Scatter plot between gene expression in the iPS state and differentiation efficiency for the X chromosome gene *ZDHHC9*.

Previous studies have demonstrated that iPSC lines vary in their capacity to differentiate^21^. As a measure of differentiation efficiency in our experiments, we used average pseudotime on day3, and observed significant variation across cell lines, which was consistent across replicate differentiations of the same cell line (Fig. 5a). Exploiting the scale of our study and the pooled experimental design, we set out to identify genetic and molecular markers of differentiation efficiency that are accessible prior to differentiation (Methods).

First, we considered the set of 4422 eQTL lead variants at any of the three developmental stages and tested each variant for association with differentiation efficiency (using a linear mixed model; Methods). This only identified a single significant association, with the eQTL variant for *DPH3* (FDR 10%, Supplementary Data 12). We performed an additional set of differentiations in HipSci iPSC lines derived from individuals that were not part of the variant discovery, selected based on genotype at this variant (*n* = 20). Differentiation efficiency was measured by the percentage of CXCR4+ cells on day3. While the direction of effect was consistent, the association was not statistically significant (*P* = 0.24, Student’s *t*-test), likely reflecting low power at this sample size. We conclude that larger sample sizes will be required to definitively reveal genetic determinants of in vitro differentiation efficiency.

We next asked whether levels of gene expression at the iPSC stage could represent molecular markers of differentiation efficiency. This revealed 38 associations (FDR 10%, 11,231 genes tested; Supplementary Data 13), 9 of which were also observed when using independent bulk RNA-seq data from the same cell lines (replication defined as nominal *P* < 0.05; Supplementary Data 13; Methods). We note that expression of these marker genes is largely orthogonal to pseudotime itself (Supplementary Fig. 18). As an example, the expression of *ZDHHC9* in iPSCs was negatively associated with differentiation efficiency (Fig. 5b). Furthermore, *ZDHHC9* is one of 17 differentiation-associated genes located on the X chromosome, reflecting a significant enrichment of X chromosome genes (24.5-fold enrichment, *P* = 8 × 10^−16^, Fisher’s exact test). Higher expression of these genes was associated with reduced differentiation efficiency (Fig. 5b; Methods). The majority of these associations persisted when limiting the analysis to female lines (14/17 at *P* < 0.05), indicating variation beyond differences between sexes. These results are consistent with previous observations that X chromosome reactivation is a marker of poor differentiation capacity of iPSCs in general^22,23^. We did not identify any striking patterns other than the reported overrepresentation of chromosome X genes, partly due to a small sample size.

## Discussion

Our map of early endoderm differentiation across 125 individuals offers a unique and powerful tool for interrogating the role of genetic heterogeneity in early human development. It characterises the effects of common genetic variants on gene expression in mesendoderm and definitive endoderm cells, providing the first eQTL maps at these key developmental stages. We exploited this resource to identify hundreds of eQTL that act at tightly-defined time points during early differentiation, and in specific cellular states, thus fully utilising the power of single-cell transcriptomics.

These results illustrate a difference between bulk and single-cell transcriptomics, as applied to eQTL mapping: the trade-off between statistical power and cellular resolution. In our analysis of iPSC, bulk transcriptomes provided higher statistical power for discovery of eQTL. However, as we have demonstrated, a single-cell approach allows detailed annotation of changing eQTL effects across heterogeneous cell types and cell states, with the ability to better interpret the context-specific role of individual genetic variants. As single-cell approaches are extended to more disease-relevant tissues and cell types, this may provide important clues on the causal role of genetic variants in disease. The single-cell technology employed also has implications for what can be assessed. While we found that similar eQTL signals could be detected with both Smart-seq2 and 10x approaches, the full-length transcripts of Smart-seq2 allowed quantification of ASE, which is not possible with the 3′ fragments sequenced using the 10x protocol.

A further advantage of the application of single-cell transcriptomics in this study was to enable the pooling strategy. While the feasibility of pooling samples has previously been demonstrated for peripheral blood mononuclear cells^8^, we have extended this to cell lines differentiated together in culture. This provided higher throughput, and enabled the characterisation of intrinsic line-to-line variation in differentiation efficiency in a controlled setting. While the endoderm differentiation protocol considered here is short and efficient, other protocols (e.g., to generate neurons^24^) are much more challenging, making a pooling strategy useful for scaling up these protocols to population-scale.

Although we have considered replicates of the same line across different experimental pools, our study is based on a single iPS line per donor. In the future, experimental designs may consider multiple lines per donor, which, however, would require a different barcoding scheme as these cannot be discriminated using genetic barcodes. As a result of this experimental design, we cannot definitively distinguish between donor and line effects.

In summary, our results demonstrate the power of combining iPSC line pooling and scRNA-seq to investigate development and genetics in vitro. Sorting of cells by state allows the context-specificity of eQTL to be probed in detail across many axes of cellular variation. The scRNA-seq data also provide a rich description of the progress of differentiation across time in different cell lines. Application of this approach in other contexts will characterise the genetic component of differentiation across the spectrum of human development.

## Methods

### Pooled scRNA-seq profiling during endoderm differentiation

A total of 126 pluripotent stem cell (iPSC) lines derived from 125 donors as part of the HipSci project were considered for analysis (see Supplementary Data 2 for demographic information). HipSci cell lines were derived from consented research volunteers recruited from the NIHR Cambridge BioResource, approved under ethics for iPS cell derivation (REC 09/H0304/77, V2 04/01/2013, and revised consent REC 09/H0304/77, V3 15/03/2013). Batches of 4-6 cell lines were co-cultured and grown as a mixed population for a total of 28 experiments, in 12 well plates. This scales up our previous work assaying single-cell transcriptomes of individual cell lines^25^. Cells were harvested immediately prior to the initiation of differentiation (day0; iPSCs), and at time points 1, 2, and 3 days post differentiation initiation (day1, day2, day3). Subsequently, single cells were sorted into 384 well plates. Cells were processed using Smart-seq2 for scRNA-seq with parallel FACS analysis of the markers TRA-1-60 and CXCR4 being performed for each cell. A subset of cell lines were assayed in more than one experiment (33 donors; Supplementary Data 1, 2; Supplementary Fig. 2). In addition to the differentiation of pools of cell lines by co-culture for scRNA-seq, cell lines were also differentiated individually and assayed by FACS for the percentage of CXCR4 + cells on day3, following the same protocol, in order to validate the genetic association of the *DPH3* eQTL variant with differentiation. These were performed as separate experiments.

### Cell culture for maintenance and differentiation

Human iPSC lines were thawed for differentiation and maintained in Essential 8 (E8) media (LifeTech) on vitronectin (StemCell Technologies, #07180) coated Corning plates according to the manufacturer’s instructions. Cells were passaged at least twice after thawing and always 3–4 days before plating for differentiation to ensure all the cell lines in each experiment were growing at a similar rate prior to differentiation. Gelatine/MEF coated plates were prepared 24–48 h before plating for differentiation by incubating plates with 0.1% gelatine for 20 min at room temp. The gelatine was then aspirated and plates were incubated in MEF medium overnight at 37 °C. Immediately prior to plating cells, plates were washed once with D-PBS to remove any residual MEF medium. To plate for endoderm differentiation, cells were washed once with D-PBS and dissociated using StemPro Accutase (Life Technologies, A1110501) at 37 °C for 3 - 5 min. Colonies were fully dissociated through gentle pipetting. Cells were resuspended in MEF medium, passed through a 40 µm cell strainer, and pelleted gently by centrifuging at 300*g* for 5 min. Cells were re-suspended in E8 media and plated at a density of 15,000 cells per cm^2^ on gelatin/MEF coated plates ^6,26^ in the presence of 10 µM Rock inhibitor—Y27632 (Sigma, #Y0503 - 5 mg). Media was replaced with fresh E8 free of Rock inhibitor every 24 h post plating. Differentiation into definitive endoderm commenced 72 h post plating. Cells were washed 1x gently with D-PBS to remove residual E8. Cells were then incubated in CDM-PVA containing 100 ng/mL ActivinA (made in house), 80 ng/mL FGF2 (made in house), 10 ng/mL BMP4 (R&D systems, #314-BP-050), 10 µm Ly294002 (Promega, #V1201), and 3 µM CHIR99201 (Selleckchem, #S1263) for 24 h (Day 1). After 24 h, the day 1 media was replaced with CDM-PVA containing 100 ng/mL ActivinA, 80 ng/mL FGF2, 10 ng/mL BMP4, and 10 µm Ly294002 for another 24 h (Day 2). Day 2 media was then replaced with RPMI/B27 containing 100 ng/mL ActivinA and 80 ng/mL FGF2 for another 24 h (Day 3) ^6^. The overall efficiency of the differentiation protocol was validated using reference lines with good and poor differentiation capacity, respectively (Supplementary Fig. 19). Please refer to the table below (Supplementary Table 3) for compositions of gelatine, MEF media, CDM-PVA, and RPMI/B27. All media was filtered through 0.22 µm filters prior to use.

### Single cell preparation and sorting for scRNAseq

Cells were dissociated into single cells using Accutase and washed once with MEF medium as described above when plating cells for differentiation. For all subsequent steps, cells were kept on ice to avoid degradation. Approximately 1 × 10^6^ cells were re-suspended in PBS + 2% BSA + 2 mM EDTA (FACS buffer); BSA and PBS were nuclease-free. For staining of cell surface markers, 1 × 10^6^ cells were re-suspended in 100 µL of ice-cold FACS buffer containing 20 µL anti-Tra-1-60 antibody (BD Pharmingen, BD560380) and 5 µL of anti-CXCR4 antibody (eBioscience 12-9999-42), and were placed on ice for 30 min. Cells were protected from light during staining and all subsequent steps. Cells were washed with 5 mL of FACS buffer, passed through a 35 µm filter to remove clumps, and re-suspended in 300 µL of FACS buffer for live cell sorting on the BD Influx Cell Sorter (BD Biosciences). Live/dead marker 7AAD (eBioscience 00-6993) was added immediately prior to analysis at a concentration of 2 µL/mL and only living cells were considered when determining differentiation capacities. Living cells stained with 7AAD but not TRA-1-60 or CXCR4 were used as gating controls. Data for TRA-1-60 and CXCR4 staining were available for 31,724 cells, of the total 36,044. Single-cell transcriptomes of sorted cells were assayed as follows: reverse transcription and cDNA amplification was performed according to the SmartSeq2 protocol ^7^, and library preparation was performed using an Illumina Nextera kit. Samples were sequenced using paired-end 75 bp reads on an Illumina HiSeq 2500 machine (one lane of sequencing per 384 well plate).

### Immunofluorescence staining

All volumes below are given per well. Cells were grown in 12 well plates and fixed at 4 °C with 500 μL of 4% paraformaldehyde (PFA; VWR, #43368.9 M) solution immediately after removing the culture medium. They were then washed three times in D-PBS to remove the fixative. Unspecific binding was blocked by incubating cells in 500 μL of PBST (0.1% Triton X-100 in D-PBS) containing 10% donkey serum (AbD Serotec, #C06SB) for 60 min at room temperature. Cells were then incubated overnight at 4 °C with 300 μL of primary antibodies diluted in PBST containing 1% donkey serum. Cells were next washed three times with D-PBS to remove unbound primary antibodies and then incubated with 300 μL of secondary antibodies diluted in PBST containing 1% donkey serum in for 1 h at room temperature. Unbound antibodies were removed by three 5 min washes in D-PBS. 4′,6-Diamidino-2- phenylindole dihydrochloride (DAPI; Sigma-Aldrich, #D-8417) at a dilution of 1:1000 was added to the first wash. Antibodies used for immunostaining and their dilutions are listed in Supplementary Table 4.

### Fluorescence activated cell sorting (FACS) analysis

Cells were washed twice in D-PBS and incubated in Accutase for 5 min at 37 °C. The Accutase was neutralised by adding double its volume of 5% FBS diluted in D-PBS and the cells were fully dissociated by gentle pipetting. Cells were washed twice in D-PBS then fixed by re-suspending in 500 μL of 4% PFA solution diluted in D-PBS for 20 min on ice. Fixed cells were washed twice in D-PBS + 2% FBS and then stored at 4 °C in BD stain buffer (BD Pharmingen Cat # 554656) for up to a week prior to analysis. For staining of intracellular markers, cells were permeabilized in 500 μL of D-PBS containing 1% Saponin (Sigma-Aldrich, #47036-50G-F) for 30 min at room temperature. Cells were then incubated for 1 h at room temperature with Goat anti-human Sox17 (R&D, #AF1924, [2 mg/mL]) at a dilution of 1:50 in 100 μL of Staining Solution (1% Saponin and 5% FBS in D-PBS). Cells were then washed twice with 1 ml of Staining Solution per wash. To visualise Sox17 staining, cells were next incubated with Donkey anti-goat 647 (Invitrogen, A21447) at a dilution of 1:1000 in 100 μL of Staining Solution for 30 min at room temperature. Cells were washed twice again with 1 mL of Staining Solution per wash and re-suspended in 200 μL of 2% FBS diluted in D-PBS prior to analysis. Analyses were performed using a BD LRSFortessa cell analyser (BD Biosciences). All flow cytometry experiments were gated using unstained cells. Data analyses were performed on FlowJo.

### RNA isolation and RT-quantitative (q)PCR

For total RNA isolation, three wells were individually harvested per condition to obtain biological replicates. The GenElute Mammalian Total RNA miniprep kit (Merck, #RTN350) and On-Column DNase I Digestion Set (Merck, #DNASE70) were used to isolate total RNA and remove contaminating genomic DNA according to the manufacturer’s instructions. To generate cDNA, 500 ng of RNA, random primer (Promega, #C1181) and dNTP (Promega, #U1511) were first incubated for 5 min at 65 °C then quickly chilled on ice to denature the RNA and primer. RNaseOUT Recombinant Ribonuclease Inhibitor (Invitrogen, # 10777019) and SuperScript II Reverse Transcriptase (Invitrogen, #18064014) were then added and the tube was incubated for 10 min at 25 °C for the primer annealing step, 50 min at 42 °C for the extension step, and finally 15 min at 70 °C for inactivation of the enzyme. The resulting cDNA was diluted to a final volume of 600 μL with nuclease-free water prior to use for RT-qPCR. RT-qPCR master mix was prepared using Sensi Mix Sybr Low Rox Kit (Bioline, #QT625-20). RT-qPCR reactions were performed using the Mx3005P system (Stratagene) according to the manufacturer’s instructions. Samples were run in technical triplicates and normalised to PBGD. Gene-specific primers are listed in Supplementary Table 5.

### Genotyping

All iPSC lines were genotyped using the Illumina HumanCoreExome-12 Beadchip and the genotypes were called using GenomeStudio (Illumina, CA, USA). Genotypes were then phased using SHAPEIT v2.r790^27^ and imputed, per sample, using IMPUTE2 v2.3.1^28^. Imputation was performed based on a joint reference panel of haplotypes derived from the UK10K cohorts and 1000 Genomes Phase 1 data^27,29^. Single-sample VCFs were merged and subsequent QC was performed using Genotype Harmonizer^30^ and BCFtools. Variants with INFO score lower than 0.4 were excluded from further analysis. More information on the genotyping and imputation procedure can be found in Kilpinen et al.^1^.

### Demultiplexing donors from pooled experiments

Assignment of cells to donors was performed using Cardelino^9^. Briefly, Cardelino estimates the posterior probability of a cell originating from a given donor based on common variants in scRNA-seq reads, while employing a beta binomial-based Bayesian approach to account for technical factors (e.g. differences in read depth, allelic drop-out, and sequencing accuracy). For this assignment step, we considered a larger set of *n* = 490 HipSci lines with genotype information, which included the 126 lines used in this study. A cell was assigned to a donor if the model identified the match with posterior probability >0.9, requiring a minimum of 10 informative variants for assignment. Cells for which the donor identification was not successful were not considered further. Across the full dataset 99% of cells that passed RNA QC steps (below) were successfully assigned to a donor.

### scRNA-seq quality control and processing

Adaptors of raw scRNA-seq reads were trimmed using Trim Galore!^31–33^, using default settings. Trimmed reads were mapped to the human reference genome build 37 using STAR^34^ (version: 020201) in two-pass alignment mode, using the default settings proposed by the ENCODE consortium (STAR manual). Gene-level expression quantification was performed using Salmon^35^ (version: 0.8.2), using the “--seqBias”, “--gcBias” and “VBOpt” options using ENSEMBL transcripts (built 75)^36^. Transcript-level expression values were summarised at a gene level (estimated counts per million (CPM)) and quality control of scRNA-seq data was performed with the *scater* Bioconductor package in R^37^. Cells were retained for downstream analyses if they had at least 50,000 counts from endogenous genes, at least 5000 genes with non-zero expression, less than 90% of counts came from the 100 highest-expressed genes, less than 15% of reads mapping to mitochondrial (MT) genes, they had a Salmon mapping rate of at least 60%, and if the cell was successfully assigned to a donor (Supplementary Fig. 20). Dead cells as identified based on 7AAD staining were discarded. Size factor normalisation of counts was performed using the *scran* Bioconductor package in R^38^. Expressed genes with an HGNC symbol were retained for analysis, where expressed genes in each batch of samples were defined based on (i) raw count >100 in at least one cell prior to QC and (ii) average log2(CPM+1) >1 after QC. Normalised CPM data were log transformed (log2(CPM+1)) for all downstream analyses. The joint dataset was investigated for outlying cell lines or experimental batches, which identified no clear groups of outlying cells (Supplementary Fig. 21, 22).

As a final QC assessment, we considered possible differences between cell lines from healthy and diseased donors. In particular, a subset of 11 cell lines were derived from neonatal diabetes patients, and differentiated together with cell lines from healthy donors across 7 experiments (out of 28). There was no detectable difference in differentiation capacity between healthy and neonatal diabetes lines in these experiments (*P* > 0.05), and cells from both sets of donors overlapped in principal component space (Supplementary Fig. 23). Thus, we included cells from all donors in our analyses irrespective of disease state.

The final merged and QC’ed dataset consisted of 36,044 cells with expression profiles for 11,231 genes (Supplementary Figs. 2 and 5).

### Bulk RNA-Seq quality control and processing

Raw RNA-seq data for 546 HipSci cell-lines were obtained from the ENA project: ERP007111 and EGA projects: EGAS00001001137 and EGAS00001000593 (see Data availability). CRAM files were merged per cell-line and converted to FASTQ format. Processing of the merged FASTQ files was matched to the single cell processing, as described above. Samples with low quality RNA-seq were discarded based on the following criteria: lines with less than 2 billion bases aligned, with less than 30% coding bases, or with a duplication rate higher than 75%. This resulted in 540 lines for analysis, 108 of which had matched (day0) single cell RNA-seq data available.

Gene-level expression levels were quantified using Salmon, analogously to the alignment, as described for the single cells. Gene expression profiles were normalised using *scran*, to match the single cell data processing, and the *scran* normalised CPM data is log transformed (log2(CPM+1)).

### Variance component analysis

Variance component analysis was performed, per gene, by fitting a random effects model using LIMIX^39^ to the gene’s expression profiles across cells. To reduce computational cost, we considered a random subset of 5000 cells. The experiment, day of collection, and cell line identity were each included as random effects. Full variance component results for all genes are provided in Supplementary Data 14.

### Highly variable genes

The top highly variable genes were computed using *scran*’s *trendVar* and *decomposeVar* functions, using a design matrix to correct for the differentiation experiment-specific effects (i.e., treating each experiment as a different batch). At FDR < 1%, this identified 4546 highly variable genes.

### Pseudotime definition

We used the first principal component calculated based on the top 500 highly variable genes in our set to represent differentiation pseudotime. This component was linearly re-scaled to take values between 0 (the minimum value observed for any cell) and 1 (the highest value observed). For comparison, we considered three alternative methods for defining pseudo time:

(i) We considered diffusion pseudotime (DPT)^40^ (Supplementary Fig. 7A). The underlying diffusion map was generated using 15 nearest neighbours and with gene expression represented by the first 20 PCs across the top 500 most highly variable genes. DPT analysis was carried out using the default settings with Scanpy v1.2.2^41^. There was a Pearson correlation of 0.82 between DPT and the pseudotime definition we used.
(ii) We considered calculating pseudotime by projecting each cell on to the principal curve of the first two principal components of the top 500 most highly variable genes (Supplementary Fig. 7B). Principal curve analysis was performed using the R package princurve^42^. There was a Pearson correlation of 0.86 between the principal curve pseudotime and the pseudotime definition we used.
(iii) We considered representing pseudotime by the mean expression of the differentiation co-expression module. This gene cluster was enriched for GO terms associated with differentiation including ‘anatomical structure morphogenesis’ (GO:0009653), ‘anterior/posterior pattern specification’ (GO:0009952), and ‘response to BMP’ (GO:0071772) (Supplementary Table 2, Supplementary Data 6**;** Supplementary Fig. 7C). There was a Pearson correlation of 0.64 between the differentiation co-expression module and the pseudotime definition we used. The lower concordance between pseudotime and this module is consistent with the limited set of genes included—the coexpression module only includes genes upregulated during differentiation, and therefore uses no information from changes in expression of pluripotency-associated genes.

### Assignment of cells to developmental stages

The stage labels post iPSC (mesendo and defendo) were defined using a combination of differentiation stages obtained using the single-cell defined pseudotime and knowledge based on canonical marker genes. Cells were assigned to the mesendo stage if they were collected at day1 or day2, and had pseudotime values between 0.15 and 0.5, corresponding to a pseudotime window around the peak expression of Brachyury (*T*), a marker of mesendoderm (Supplementary Fig. 8A). Cells were assigned to the defendo stage if they were collected at day2 or day3, and had pseudotime values higher than 0.7, corresponding to a pseudotime window with maximal expression of *GATA6*, a marker of definitive endoderm (Supplementary Fig. 8B). Cells with intermediate pseudotime (between 0.5 and 0.7) mostly came from day2, and were not assigned to any stage for the purposes of the initial stage QTL mapping (results shown in Fig. 2, Supplementary Table 1). Overall, we assign 28,971 (80%) cells to any of the stages (iPSC, mesendo, defendo).

### *cis* eQTL mapping

A consistent eQTL mapping strategy was applied to bulk RNA-seq expression and expression traits derived from scRNA-seq. We considered common variants (minor allele frequency >5%) within a *cis-*region spanning 250 kb up- and downstream of the gene body for *cis* QTL analysis. Association tests were performed using a linear mixed model (LMM), accounting for population structure and sample repeat structure (see below) as random effects (using a kinship matrix estimated using PLINK^43^), with no observed confounding between population structure and experimental batch (Supplementary Fig. 24). All models were fitted using LIMIX^39^. The values of all features were standardised and the significance was tested using a likelihood ratio test (LRT). To adjust for experimental batch effects across samples, we included the first 10 principal components calculated on the expression values in the model as covariates. These batch effects usually affect the expression of many genes, and therefore are detectable in the principal components of expression. Furthermore, such global batch effects are orthogonal to the effects of a single cis regulatory variant on the expression of one gene. This approach is similar to that applied in conventional bulk eQTL analyses^1^. In order to adjust for multiple testing, we used an approximate permutation scheme, analogous to the approach proposed in^44^. Briefly, for each gene, we generated 1000 permutations of the genotypes while keeping covariates, kinship, and expression values fixed. We then adjusted for multiple testing using this empirical null distribution. To control for multiple testing across genes, we then applied the Storey procedure^45^. Genes with significant eQTL were reported at an FDR < 10%.

### Mapping cis eQTL across three stages of differentiation

To map eQTL based on scRNA-seq profiles, we quantified average gene expression profiles (log2(CPM+1)) across cells for each (donor, day of collection, experiment) combination. This approach retains differences across experiments and days, for cells from the same donor, and is enabled by the pooled experimental design. Accounting for population structure using a kinship matrix is especially important in this context, since aggregated expression values for the same donor from different experiments are essentially replicates and hence genetically identical. We separately mapped eQTL for each differentiation stage (i.e. iPSC, mesendo, defendo), yielding 1833 (10,840 tested), 1702 (10,924 tested) and 1342 (10,901 tested) genes with an eQTL respectively (FDR < 10%). eQTL results are provided in Supplementary Data 3).

For comparison, we performed analogous QTL analyses using all cells from day1, and day3 instead of the pseudotime-based differentiation stages. This approach resulted in 1181 (10,787 tested) and 631 (10,765 tested) genes with an eQTL at day 1 and 3 respectively (Supplementary Table 1).

### Mapping dynamic eQTL (visualisation purposes only)

We performed eQTL mapping across a sliding window on pseudotime, considering bins that contain 25% of all cells, sliding along the pseudotime by a step of 2.5% of cells (Fig. 3a, top middle panel). Similarly to the approach taken for eQTL analysis in individual differentiation stages, expression values are averaged by (donor, day, experiment) combinations, within each window.

### Mapping *cis* eQTL in iPSCs with bulk RNA-seq

To perform *cis*-eQTL mapping in the bulk RNA-seq data, we considered cell lines that had been used to map iPSC eQTL from the scRNA-seq data (bulk data was available for 108 donors out of the 112 day0 single cell donors), and tested the same set of genes. This yielded 2908 significant genes at an FDR of 10% (out of 10,736 genes tested).

To compare the iPSC eQTL maps derived from bulk and single-cell RNA-seq data, we assessed the nominal significance (*P* < 0.05) as well as the consistent direction of effect of single-cell iPSC eQTL lead variants (top variant per gene) in the full set of results from the bulk iPSC eQTL analysis and vice versa.

### SNP tagging

We used LD tagging to account for linkage disequilibrium (LD) effects that might cause false positive lead switches and to identify links between GWAS implicated variants and eQTL. To this end, we calculated the LD between lead eQTL variants and either GWAS variants or other eQTL lead variants, using both the 1000 genomes phase3 reference panel and the HipSci dataset to calculate LD between SNPs, taking the union of both sets.

### Lead switching event quantification

Lead switching events were defined as two or three distinct variants that were identified at distinct differentiation stages, found to be significantly associated (FDR < 10%) with the same genes, and that were not in LD (*r*^2 < ^0.2).

### GWAS tagging

We performed GWAS tagging using an LD threshold of *r*^2^ > 0.8. We considered all GWAS variants from the GWAS catalogue as available as part of ENSEMBL 94^46^, for all traits and diseases. This analysis was restricted to variants that reached genome-wide significance (*P* < 5e-8) for any of the traits.

### Allele-specific expression quantification

Duplicated reads were removed from the STAR alignments using Picard Tools (http://broadinstitute.github.io/picard). ASE was quantified at the gene level relative to a heterozygous eQTL lead variant. As a result, for a given eQTL, ASE was only quantified across cells from donors heterozygous for that eQTL variant. This was done following five steps (see Supplementary Fig. 25 for a worked example of one gene in one cell): (1) ASE counts were obtained using GATK tools v3.7 in ASEReadCounter mode, with the settings “-minDepth 1 -minMappingQuality 10 -minBaseQuality 2 -rf DuplicateRead”. ASE of a SNP in a given cell was quantified if (i) the cell was heterozygous for that SNP, based on the known donor genotypes, and (ii) the SNP was located in an exonic region (ENSEMBL 75 annotation, as above). The output from GATK tools gives the number of reads mapping to the alternative and reference alleles for each heterozygous SNP in each cell. (2) For each cell, ASE quantifications for each SNP were converted from “alternative allele reads” to “chrB allele reads” using the known phase (indicated as chrA|chrB, where 0 = reference, 1 = alternative) of each SNP in each donor (e.g. for a SNP with the phase “1|0”, the alternative allele is on chrA, so the number of reads mapping to chrB = number of reference allele reads = total number of reads—number of alternative allele reads). Thus, for each cell, ASE for all SNPs was quantified relative to the genotypes of the chromosomes of that individual, rather than to “reference” or “alternative” alleles. (3) Aggregation of ASE from SNP-level to gene-level. For each gene, this was done by summing the “chrB allele reads” and “total reads” across all SNPs contained in the exons of that gene (as described in the ENSEMBL 75 GTF file). (4) Conversion of quantifications from “chrB allele reads” to “reads from the chromosome containing the alternative allele of the eQTL SNP”, again by using the available phasing information. For each eQTL (i.e. each gene-SNP pair), this provides a consistent definition of ASE across all cells heterozygous for the eQTL SNP (i.e., across cells from multiple donors). Donors that are not heterozygous at the eQTL variant of interest were not used for quantification. (5) Conversion to allelic fractions i.e. quantifications express the allelic reads as a fraction of the total number of reads.

### Mapping of dynamic and interaction eQTL using ASE

ASE quantifies the relative expression of one allele over the other. If one of these alleles is more responsive to a particular environmental factor (e.g., because of preferential transcription factor binding), then ASE is expected to vary systematically with that factor. This observation has previously been used to identify GxE interactions in gene expression across individuals^17^. Here, we applied similar concepts to single-cell RNA-seq, testing for the influence of cellular environmental factors (i.e., cellular processes) on ASE in individual cells. Importantly, these ASE tests are internally matched, as potentially confounding batch effects and technical variation affect both alleles in each cell similarly.

Three sets of tests were performed, in a linear modelling framework (Fig. 4, Supplementary Fig. 17**;** Supplementary Data 13):

(1) Testing for dynamic eQTL i.e. where ASE depends on pseudotime (“*pseudo*”). The ASE of each gene-eQTL pair was jointly tested for linear and quadratic dependence on pseudotime (two degrees of freedom likelihood ratio test), across all cells in which ASE was quantified for that pair:

1 $${\mathrm{ASE}} = {\mathrm{pseudo}} + {\mathrm{pseudo}}^2 + \varepsilon$$

(2) Pseudotime-corrected linear cellular factor test. As (1), but with each of 4 cellular factors (“*factor*”) (respiratory metabolism, sterol biosynthesis, G1/S transition and G2/M transition) and linear and quadratic pseudotime included as covariates (note that these covariates are included regardless of whether an eQTL was identified as dynamic in test (1)):

2 $${\mathrm{ASE}} = {\mathrm{pseudo}} + {\mathrm{pseudo}}^2 + {\mathrm{factor}} + \varepsilon$$

(3) Pseudotime-factor interaction test. As (2), but testing for the additional effect of (pseudotime x factor) included as a covariate:

3 $${\mathrm{ASE}} = {\mathrm{pseudo}} + {\mathrm{pseudo}}^2 + {\mathrm{factor}} + ({\mathrm{pseudo}} \times {\mathrm{factor}}) + \varepsilon$$

In each case, tests were only performed for eQTL for which ASE was quantified in at least 50 cells. Tests were performed using the statsmodels package in Python (likelihood ratio test). Multiple testing correction was performed independently for each of the three sets of tests, using Benjamini-Hochberg correction.

### Binning ASE across pseudotime

For visualising ASE as a function of pseudotime or other cellular factors, we averaged ASE across bins of 25% of cells, as done for the sliding window eQTL analysis (above). For each (eQTL x bin) combination, the mean ASE, number of cells, standard deviation, and standard error of the mean (SEM) was calculated (noting that, while each bin contains an equal number of cells, not all cells have quantified ASE for each gene). For each eQTL, to calculate the dynamics of allelic expression across pseudotime (i.e., the expression of transcripts from the ALT and REF chromosomes, as plotted in Fig. 3c), two calculations were performed. First, the mean expression of each gene across the pseudotime bins was calculated using all cells heterozygous for the eQTL SNP (i.e., the cells in which ASE was quantified). The expression of each allele in each pseudotime bin was then calculated by taking the mean ASE+/− SEM, multiplied by the mean expression of that gene (in CPM) in that bin.

### Coexpression and covariation clustering

Grouping of pseudotime-smoothed gene expression and allele-specific expression (see below) was performed by spectral clustering, as implemented by the Python scikit-learn library (Fig. 3). The negative of the Pearson correlation was used as the dissimilarity metric. A range of cluster numbers were tried, with *N* = 4 judged to be the most clusters possible before highly correlated pairs of clusters were observed.

Grouping of genes by single-cell co-expression was performed using affinity propagation^47^, as implemented by the Python scikit-learn library^48^. The Pearson correlation across all cells was used as the similarity/‘affinity’ metric. The top 8,000 highest expressed genes were included in this clustering (as judged by average expression across all cells). This generated a set of 60 co-expression clusters. GO enrichment of each cluster was performed by Fisher’s exact test in Python using GOATOOLS^49^, and results are listed in Supplementary Data 6 (FDR 10%).

Exemplar co-expression clusters were selected to represent 4 dimensions of cellular state (Fig. 4a, Supplementary Table 2): cell cycle G1/S transition (cluster 10), cell cycle G2/M transition (cluster 30), cellular respiration (cluster 0), and sterol biosynthesis (cluster 28). This selection was done according to two criteria: (1) strongest enrichment of relevant GO terms. The co-expression clusters showed the largest overrepresentation of genes for the GO terms ‘G1/S transition of mitotic cell cycle’ (GO:0000082; cluster 10), ‘G2/M transition of mitotic cell cycle’ (GO:0000086; cluster 30), ‘respiratory electron transport chain’ (GO:0022904; cluster 0), and ‘sterol biosynthetic process’ (GO:0016126; cluster 28). (2) a priori expectation of sources of cell-to-cell variation. Variation in cell cycle stage is a common feature of single-cell datasets^14^, while variation in metabolic state during iPSC differentiation is well known^50^.

### ChIP-seq experiments and data processing

ChIP-seq was performed using FUCCI-Human Embryonic Stem Cells (FUCCI-hESCs, H9 from WiCell) in a modified endoderm differentiation protocol to that used for the iPSC differentiations (see details below). Cells were grown in defined culture conditions as described previously^51^. Pluripotent cells were maintained in Chemically Defined Media with BSA (CDM-BSA) supplemented with 10 ng/mL recombinant Activin A and 12 ng/mL recombinant FGF2 (both from Dr. Marko Hyvonen, Dept. of Biochemistry, University of Cambridge) on 0.1% Gelatin and MEF media coated plates. Cells were passaged every 4–6 days with collagenase IV as clumps of 50–100 cells. The culture media was replaced 48 h after the split and then every 24 h.

The generation of FUCCI-hESC lines is based on the FUCCI system (^52,53^). hESCs were differentiated into endoderm as previously described^54^. Following FACS sorting, Early G1 (EG1) cells were collected and immediately placed into the endoderm differentiation media and time-points were collected every 24 h up to 72 h. Endoderm specification was performed in CDM with Polyvynilic acid (CDM-PVA) supplemented with 20 ng/mL FGF2, 10 µM Ly-294002 (Promega), 100 ng/mL Activin A, and 10 ng/mL BMP4 (R&D).

We performed ChIP-sequencing for various histone marks (H3K4me3, H3K27me3, H3K4me1, H3K27ac, H3K36me3) (see Supplementary Table 6 for antibodies), on two biological replicates per condition^55^. At the end of the ChIP protocol, fragments between 100 bp and 400 bp were used to prepare barcoded sequencing libraries. 10 ng of input material for each condition were also used for library preparation and later used as a control during peak calls. The libraries were generated by performing 8 PCR cycles for all samples. Equimolar amounts of each library were pooled and this multiplexed library was diluted to 8pM before sequencing using an Illumina HiSeq 2000 with 75 bp paired-end reads.

Reads were mapped to GRCh38 reference assembly using BWA^56^. Only reads with mapping quality score ≥10 and aligned to autosomal and sex chromosomes were kept for further processing. Peak calling analysis^57^ was performed using PeakRanger^58^, and only the peaks that were reproducible at an FDR of ≤0.05 in two biological replicates were selected for further processing. Peak calling was done using appropriate controls with the tool peakranger 1.18 in modes *ranger* (H3K4me3, H3K27ac; ‘-l 316 -b 200 -q 0.05’), *ccat* (H3K27me3; ‘-l 316 --win_size 1000 --win_step 100 --min_count 70 --min_score 7 -q 0.05’) and *bcp* (H3K4me1, H3K36me3; ‘-l 316’). Adjacent peak regions closer than 40 bp were merged using the BEDTools suite^59^, and those overlapping ENCODE blacklisted regions were filtered out (ENCODE Excludable Mappability Regions^60^). Finally, peaks were converted to GRCh37 coordinates using UCSC LiftOver [68].

### Identification of markers for differentiation efficiency

Differentiation efficiency for each cell line was defined as its average pseudotime across cells at day3, quantified for each experiment and unique donor. To test for associations with molecular markers, we considered stage-specific gene expression levels, again quantified for each donor and experiment (as log2(CPM+1)).

Three sets of tests were performed. In each case, models were fitted using the lme4 package in R^61^, and significance was determined by the Likelihood ratio test. The tested model was:

4 $${\mathrm{Differentiation}}\;{\mathrm{efficiency}} = {\mathrm{Marker}} + {\mathrm{Experiment}} + {\mathrm{Donor}} + \varepsilon$$

Where Experiment is a random effect grouping sets of samples from the same experiment, and Donor is a random effect grouping samples from the same donor (and cell line). Two sets of Markers were tested—genetic markers (i.e., eQTL SNPs), and expression markers (i.e. expression levels in the iPSC stage/day0), and are presented in Tables S14, S15, respectively. For genetic markers, tests were limited to the lead eQTL variant per eGene and differentiation stage.

Genetic markers were validated using data from independent differentiations of individual cell lines. Here, the percentage of CXCR4+ on day 3 (as measured by FACS) was used as a measure of differentiation efficiency, with the following model:

5 $$\% \;{\mathrm{CXCR}}4 + = {\mathrm{Marker}} + \varepsilon$$

The association identified at FDR 10%, with the eQTL variant for *DPH3*, was tested using data from other cell lines selected according to their genotype at this locus.

Expression markers were validated by comparison to bulk RNA-sequencing at the iPSC stage (day0). In particular, we tested the association between gene expression in the same cell lines, assayed in separate experiments by bulk RNA-seq of iPSCs, with differentiation efficiency in our experiments, using the model:

6 $${\mathrm{Differentiation}}\;{\mathrm{efficiency}} = {\mathrm{Marker}}\;{\mathrm{bulk}}\;{\mathrm{expression}}\;{\mathrm{in}}\;{\mathrm{iPSCs}} + \varepsilon$$

Results of the replication p-values and directions of effect are provided in Supplementary Data 13.

To evaluate whether donor sex had a significant effect on differentiation, we fit the following linear mixed model:

7 $${\mathrm{Differentiation}}\;{\mathrm{efficiency}} = {\mathrm{Sex}} + {\mathrm{Experiment}} + {\mathrm{Donor}} + \varepsilon$$

In this model Sex was modelled as a fixed effect and tested for significance using likelihood ratio test, and Experiment and Donor were modelled as random effects, as above.

### Reporting summary

Further information on research design is available in the Nature Research Reporting Summary linked to this article.

## Supplementary information

Supplementary Information

Description of Additional Supplementary Files

Supplementary Data 1

Supplementary Data 2

Supplementary Data 3

Supplementary Data 4

Supplementary Data 5

Supplementary Data 6

Supplementary Data 7

Supplementary Data 8

Supplementary Data 9

Supplementary Data 10

Supplementary Data 11

Supplementary Data 12

Supplementary Data 13

Supplementary Data 14

Reporting Summary

## Data Availability

All HipSci data can be accessed from http://www.hipsci.org. Bulk RNA-seq data are available under accession numbers: ERP007111 (ENA project) and EGAS00001001137, EGAS00001000593 (EGA projects). Single cell RNA-seq data are available under the accession numbers ERP016000 (ENA project) and EGAS00001002278, EGAD00001005741(EGA project: study ID, dataset ID). All Chip-seq data used is available at PRJNA593217. Processed single cell count data are available from Zenodo: https://zenodo.org/record/3625024#.Xil-0y2cZ0s

## Code availability

Code is available at: https://github.com/single-cell-genetics/singlecell_endodiff_paper/.
