# Publisher Correction: Single-cell RNA-sequencing of differentiating iPS cells reveals dynamic genetic effects on gene expression

**DOI:** 10.1038/s41467-020-15098-y

**Published:** 2020-03-23

**Authors:** Anna S. E. Cuomo, Daniel D. Seaton, Davis J. McCarthy, Iker Martinez, Marc Jan Bonder, Jose Garcia-Bernardo, Shradha Amatya, Pedro Madrigal, Abigail Isaacson, Florian Buettner, Andrew Knights, Kedar Nath Natarajan, HipSci Consortium, Chukwuma A. Agu, Alex Alderton, Petr Danecek, Rachel Denton, Richard Durbin, Daniel J. Gaffney, Angela Goncalves, Reena Halai, Sarah Harper, Christopher M. Kirton, Anja Kolb-Kokocinski, Andreas Leha, Shane A. McCarthy, Yasin Memari, Minal Patel, Ewan Birney, Francesco Paolo Casale, Laura Clarke, Peter W. Harrison, Helena Kilpinen, Ian Streeter, Davide Denovi, Ruta Meleckyte, Natalie Moens, Fiona M. Watt, Willem H. Ouwehand, Angus I. Lamond, Dalila Bensaddek, Philip Beales, Ludovic Vallier, John C. Marioni, Mariya Chhatriwala, Oliver Stegle

**Affiliations:** 1 European Molecular Biology Laboratory, European Bioinformatics Institute, Wellcome Genome Campus, Hinxton, CB10 1SD Cambridge UK; 2 St Vincent’s Institute of Medical Research, Fitzroy, VIC 3065 Australia; 3 Wellcome Sanger Institute, Wellcome Genome Campus, Hinxton, CB10 1SA UK; 4 Wellcome Trust—MRC Cambridge Stem Cell Institute, Anne McLaren Laboratory, University of Cambridge, Cambridge, CB2 0SZ UK; 5 Department of Surgery, University of Cambridge, Cambridge, CB2 0QQ UK; 6 Cancer Research UK Cambridge Institute, University of Cambridge, Cambridge, UK; 7 Division of Computational Genomics and Systems Genetics, German Cancer Research Center (DKFZ), 69120 Heidelberg, Germany; 8 European Molecular Biology Laboratory, Genome Biology Unit, 69117 Heidelberg, Germany; 9 Present Address: Department of Haematology, University of Cambridge, Cambridge, UK; 10 Present Address: GeneLab, AWG Multi-Omics/System Biology, NASA Ames Research Center, Moffett Field, California, USA; 11 Present Address: Danish Institute of Advanced Study (D-IAS), Functional Genomics and Metabolism Unit, University of Southern Denmark, Odense, Denmark; 12 King’s College London, Strand Ln, London, WC2R 2LS UK; 13 Department of Haematology, University of Cambridge and National Health Service Blood and Transplant, Hills Rd, Cambridge, CB2 0XY UK; 14 Centre for Gene Regulation and Expression, School of Life Sciences, University of Dundee, Dow St, Dundee, DD1 5EH UK; 15 University College London, Gower St, Bloomsbury, London, WC1E 6BT UK

**Keywords:** Functional genomics, Genetic association study, Quantitative trait loci, Induced pluripotent stem cells

Correction to: *Nature Communications* 10.1038/s41467-020-14457-z, published online 10 February 2020.

In the original version of this article, the present address of author Pedro Madrigal was incorrectly given as “GeneLab, AWG Multi-Omics/System Biology, NASA Ames Research Center, Moffett Field, California, USA”.

The correct present address of author Pedro Madrigal is “Department of Haematology, University of Cambridge, Cambridge, UK” and “GeneLab, AWG Multi-Omics/System Biology, NASA Ames Research Center, Moffett Field, California, USA”.

This has now been corrected in both the PDF and HTML versions of the Article.

