# Supplementary material for: Single-cell RNA-sequencing of differentiating iPS cells reveals dynamic genetic effects on gene expression
Source: Nat Commun. 2020 Feb 10;11:810. doi: 10.1038/s41467-020-14457-z (PMC7010688; doi:10.1038/s41467-020-14457-z)
Supplement: Supplementary file 1 — Supplementary Information [file 41467_2020_14457_MOESM1_ESM.pdf]

Supplementary Information

Single-cell RNA-sequencing of differentiating iPS cells reveals dynamic genetic effects on gene expression

Cuomo et al.

Supplementary Figures

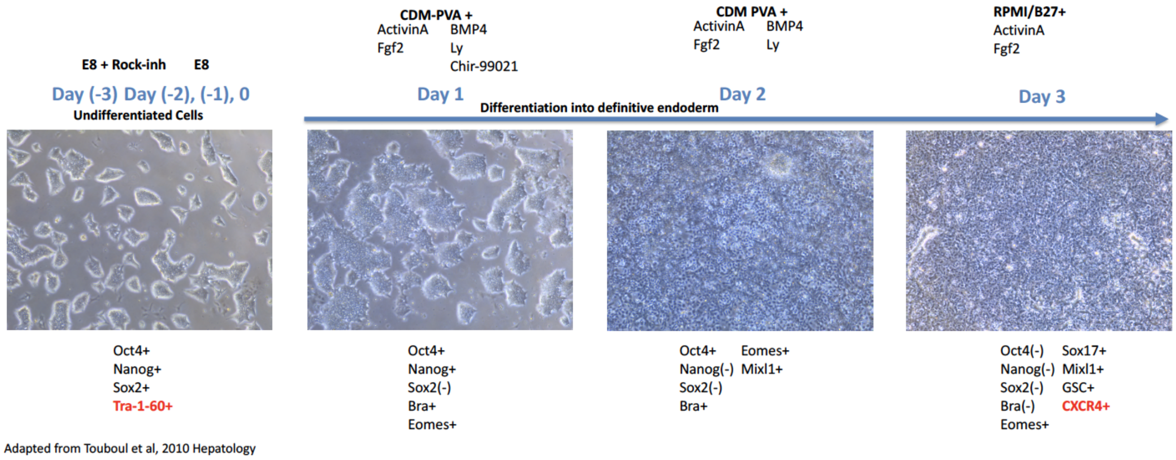

Supplementary Figure 1 | Endoderm differentiation protocol. Schematic representation of the chemically defined protocol used to initiate differentiation towards definitive endoderm (adapted from [1]). Tra-1-60 and CXCR4 are canonical cell surface markers used to sort live cells by differentiation stage.

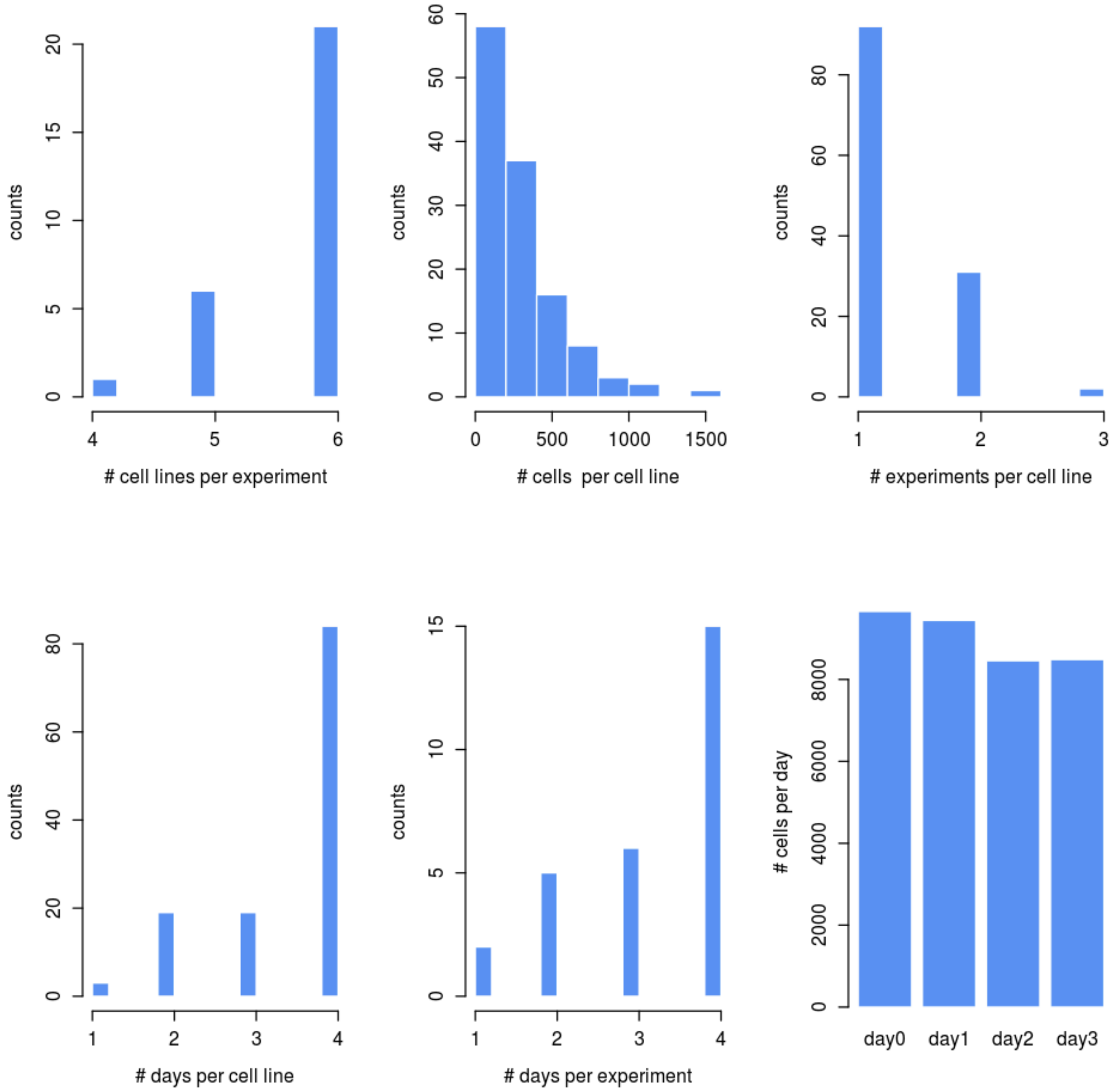

Supplementary Figure 2 | Overview of experimental metrics. Statistics for number of cells, donors, experiments, days, and combinations. Cell counts are shown after quality control.

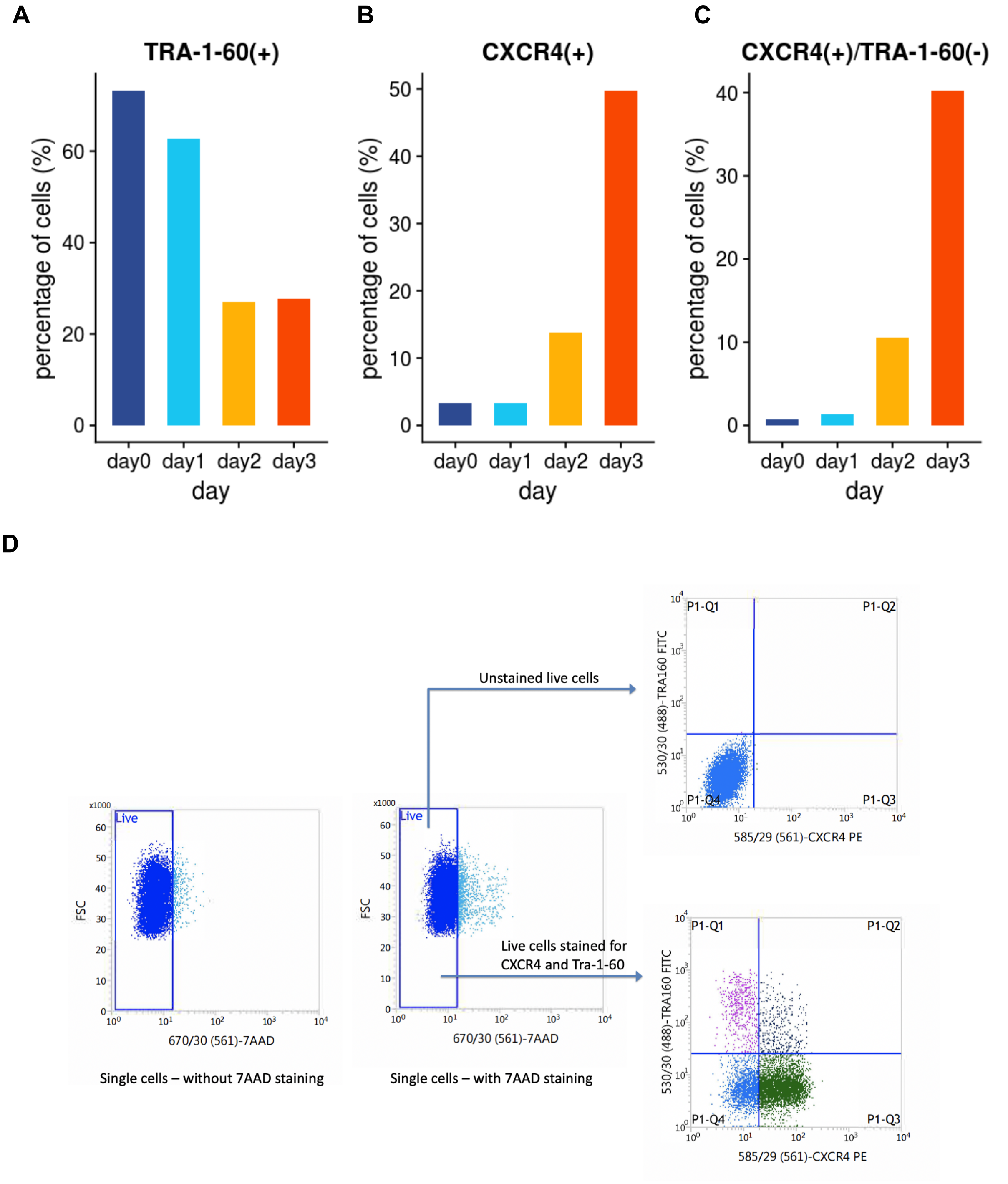

Supplementary Figure 3 | Cell surface marker expression across differentiation. Shown are the percentages of cells that are **(A)** positive for TRA-1-60, a pluripotency marker, **(B)** positive for CXCR4, a definitive endoderm marker, and **(C)** positive for CXCR4 and negative for TRA-1-60, across all cell lines and all experiments. **(D)** FACS gating strategy: First, single cells were stained with 7AAD to exclude dead cells. Unstained live cells were then used to gate for expression of Tra-1-60 and CXCR4.

A

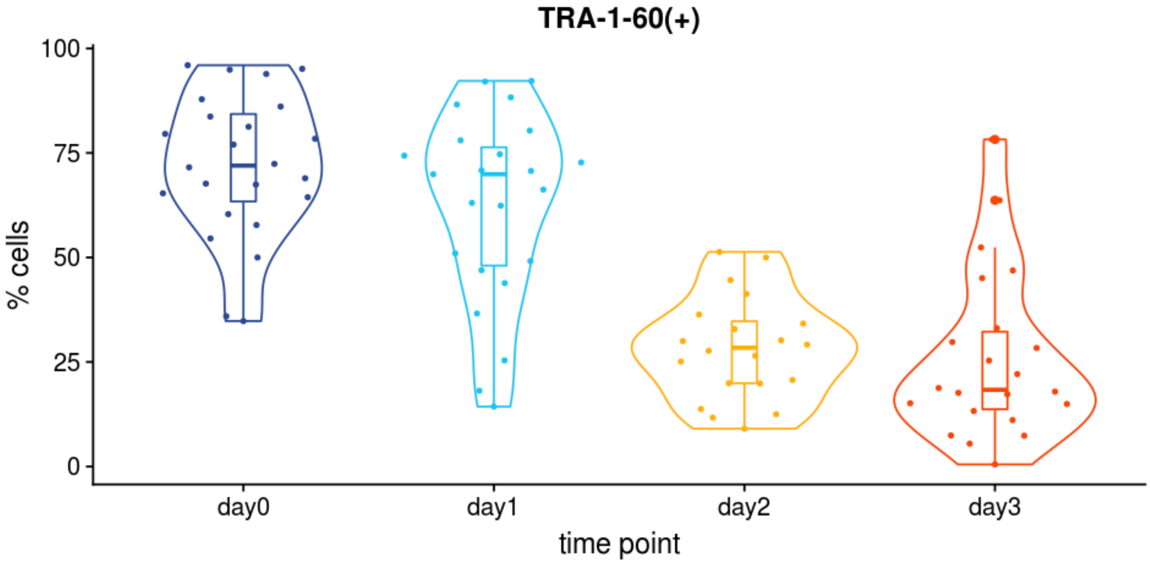

B

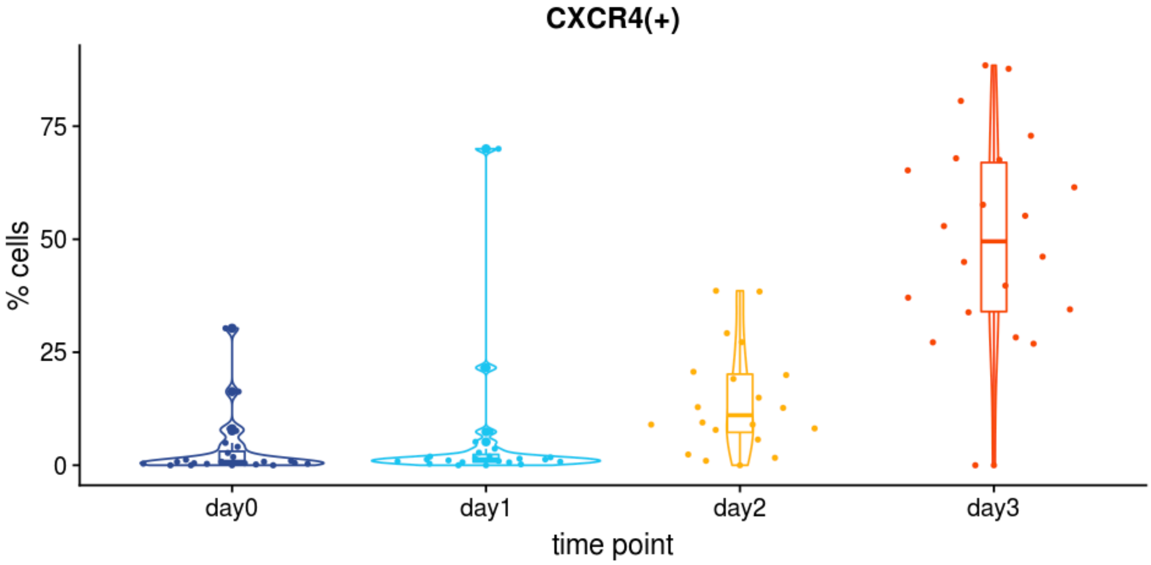

Supplementary Figure 4 | Distribution of cell surface marker expression across differentiation experiments. Shown are the percentages of cells that are (**A**) positive for TRA-1-60, a pluripotency marker, (**B**) positive for CXCR4, a definitive endoderm marker, for each experiment, by day.

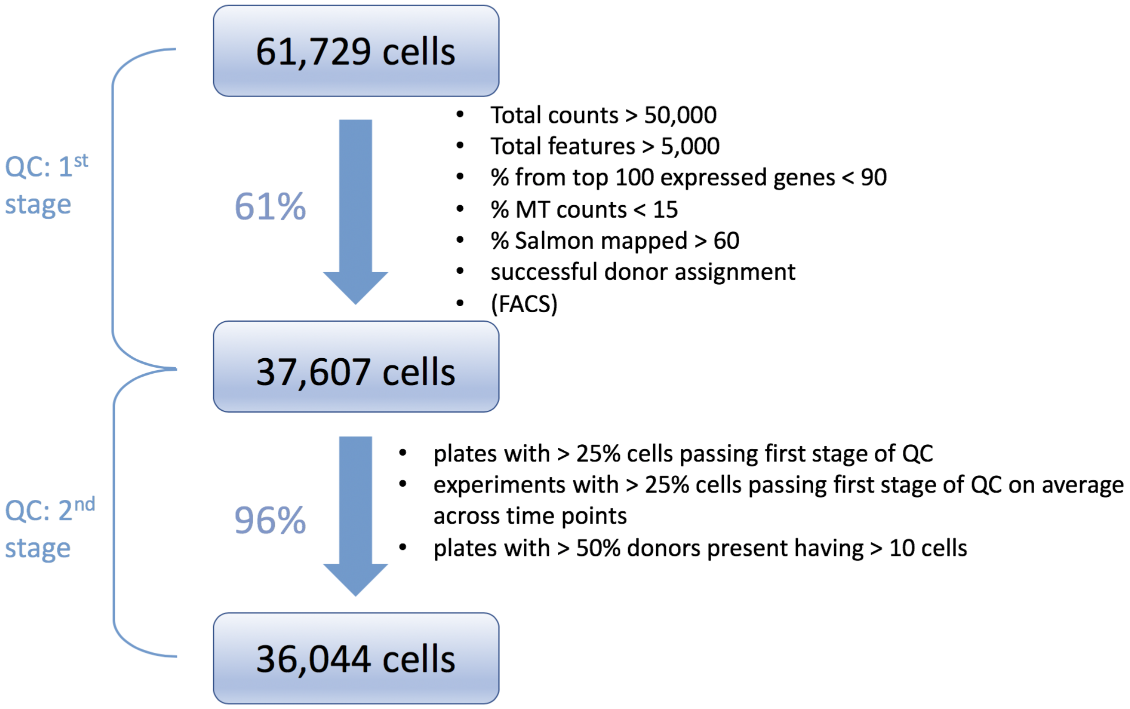

Supplementary Figure 5 | Workflow of scRNA-seq quality control. Quality control (QC) was carried out in two stages. First, QC was applied on the level of individual cells using conventional quality metrics. Second, QC was applied on the level of scRNA-seq processing plates and experimental batches, using aggregate quality metrics to retain cells from high-quality plates and experiments. Total numbers of cells before and after each QC step are shown, along with the percentage of cells retained in each QC step.

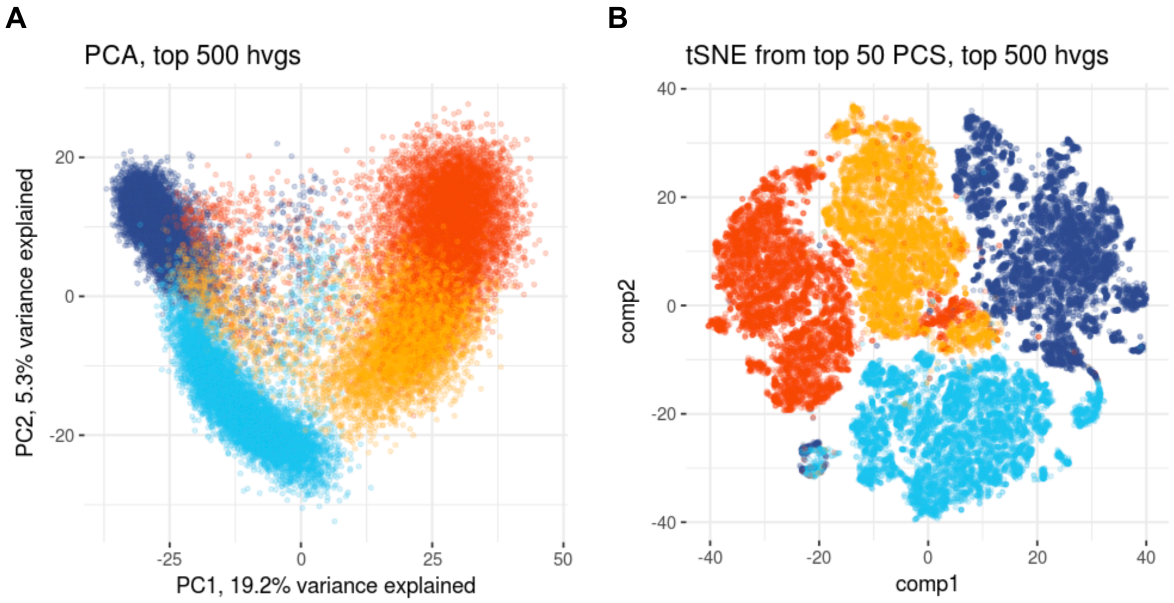

Supplementary Figure 6 | Overview of PCA and t-SNE representations of the full scRNA-seq dataset. (**A**) First two principal components (PC1 and PC2) computed on top 500 highly variable genes (**Methods**). Axes labels show the percentage of variance explained. (**B**) t-SNE plot, computed from the first 50 PCs, on the top 500 highly variable genes.

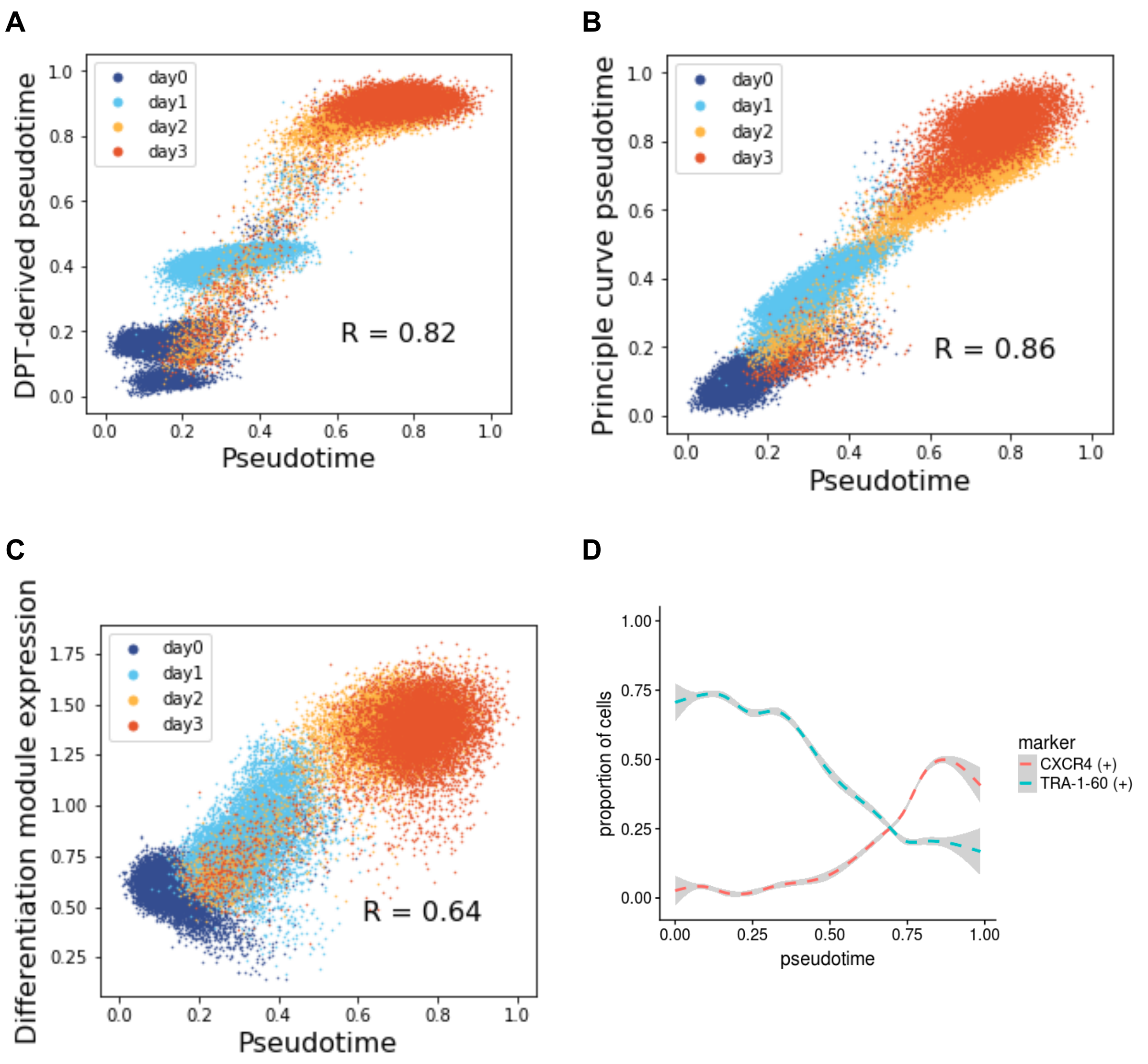

Supplementary Figure 7 | Evaluation of pseudotime definition. (**A**) Comparison of the pseudotime defined based on principal component analysis with diffusion pseudotime (DPT) [2]. The underlying diffusion map was generated using 15 nearest neighbours and with gene expression represented by the first 20 PCs across the top 500 most highly variable genes (**Methods**). (**B**) Comparison of PCA-based pseudotime with an alternative pseudotime based on projection of each cell on to a principal curve in the first two principal components of the top 500 most highly variable genes (**Methods**). (**C**) Comparison of pseudotime to the mean expression of a set of 124 co-expressed genes that are associated with cell differentiation (**Methods**). (**D**) Scatter plot of FACS markers as a function of the PCA-based pseudotime, showing expected trends.

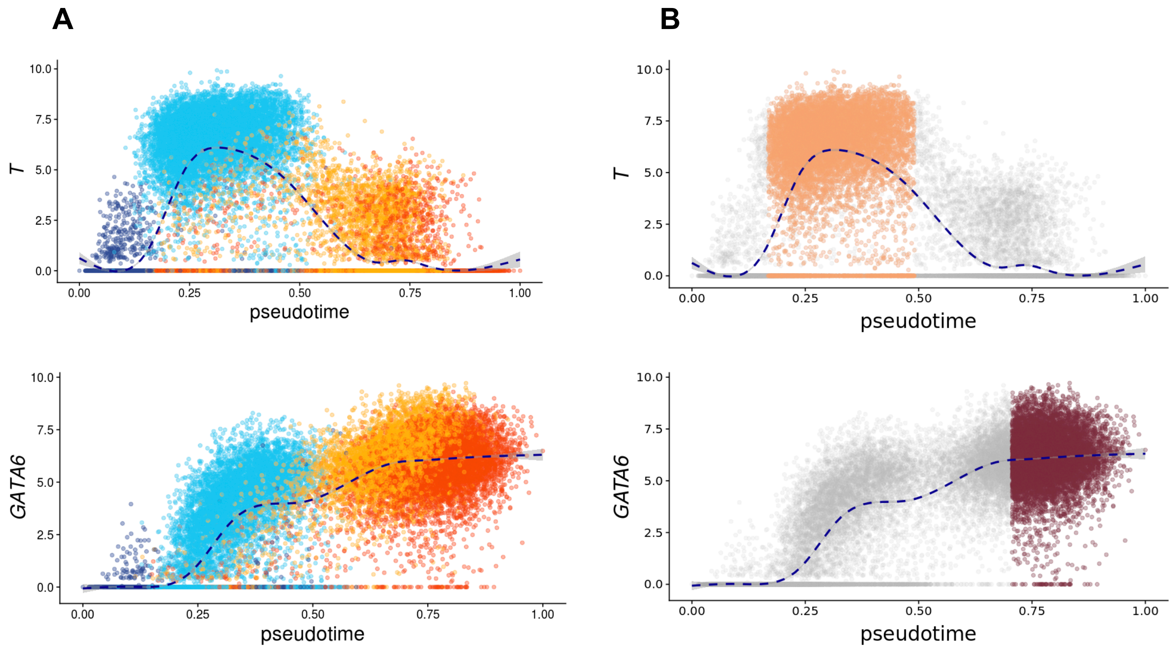

Supplementary Figure 8 | Marker gene expression in pseudotime-based developmental states. (**A**) Expression of exemplar canonical markers for mesendoderm (*T*) and definitive endoderm (*GATA6*) along pseudotime. Cells are coloured by the time point of collection, as in **Fig. 1D** (**B**) On the same plots as in **A**, cells assigned to mesendo and defendo, respectively, are highlighted (**Methods**).

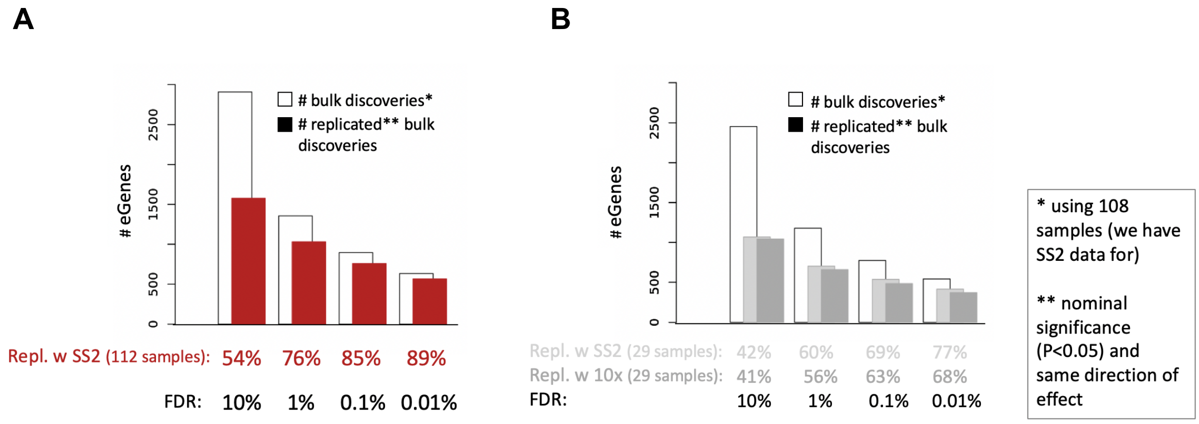

Supplementary Figure 9 | Replication of iPSC eQTL bulk eQTL using single cell across sample sizes and single cell technologies. Replication of iPSC eQTL discovered with bulk RNA-seq (108 samples), using single cell RNA-seq. The total number of bulk eQTL discovered is shown, along with the number of discoveries replicated using single cells. Replication was defined as nominal significance, at $P < 0.05$, and same direction of effect. (**A**) Replication of bulk eQTL in single-cell RNA-seq (SmartSeq2, here SS2) on same set of samples increases with significance (~55% at FDR 10%, ~90% at FDR 0.01%, 4/112 samples were not present in the bulk RNA sequencing dataset). (**B**) Replication of bulk eQTL using scRNA-seq is reduced when we reduce sample size (~40% at FDR 10%, ~70% at FDR 0.01%) but comparable across technologies (SmartSeq2, 10x Genomics), with SmartSeq2 slightly outperforming 10x. The same 29 samples are considered for both technologies.

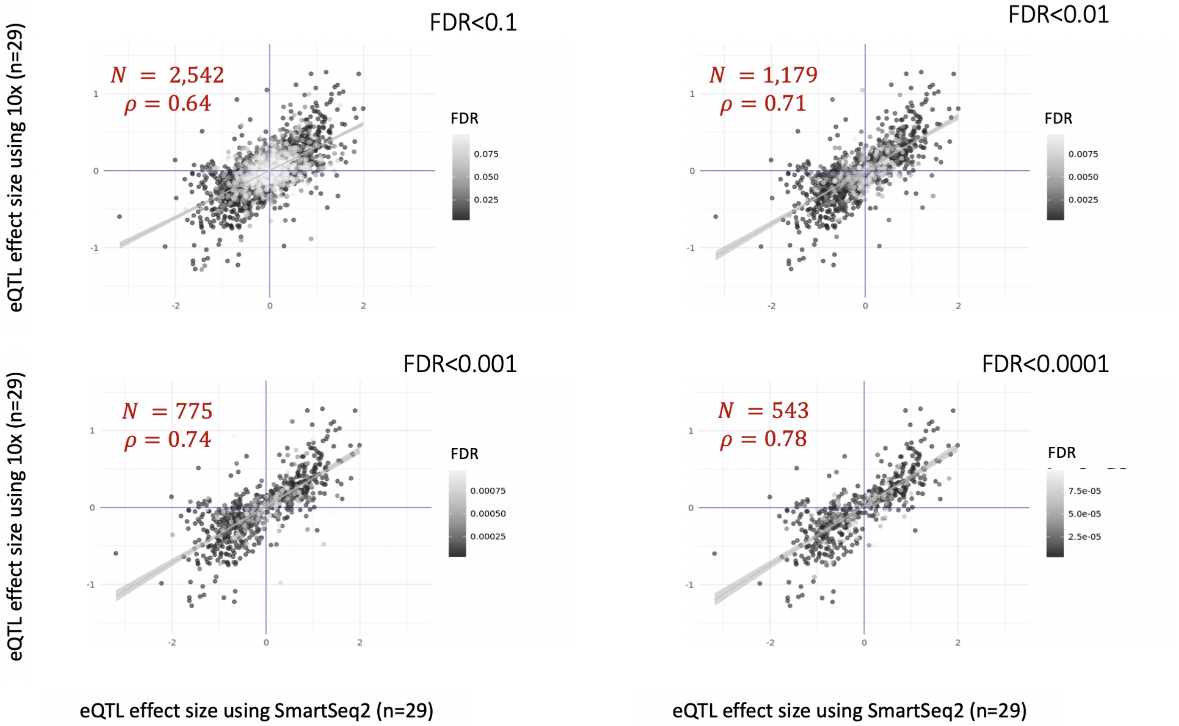

Supplementary Figure 10 | Effect size agreement between single cell technologies. Scatterplots of eQTL effect sizes obtained when testing association of iPSC eQTL discovered using bulk RNA-sequencing (108 cell lines) using SmartSeq2 (x axis) and 10x (y axis) on cells from 5 experimental batches (experiments 31, 40, 41, 43, 44; 29 cell lines in total). The number of eQTL examined and the correlation between effect sizes is indicated when we consider bulk iPSC eQTL discoveries at four different FDR thresholds (0.1, 0.01, 0.001, 0.0001).

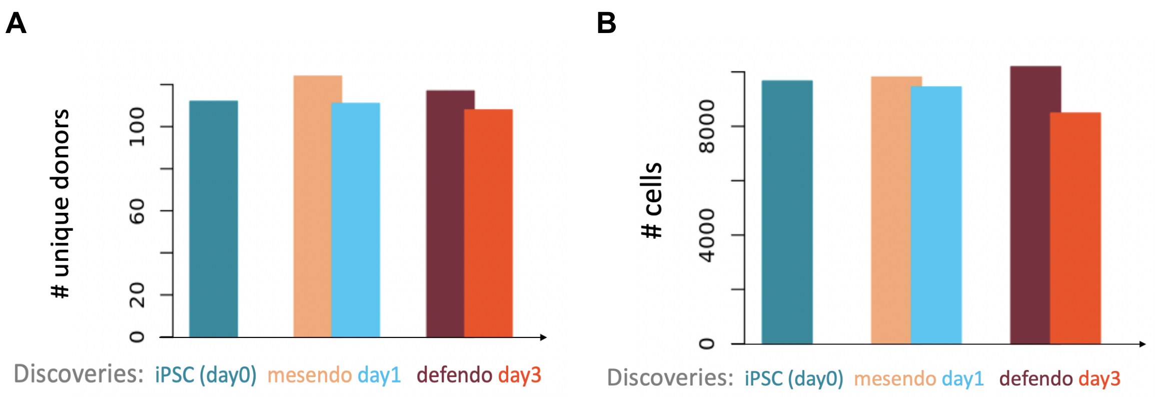

Supplementary Figure 11 | Comparison of numbers of donors and cells at each time point and each differentiation stage. Related to **Fig. 2B**. (**A**) The number of donors for which gene expression data were assayed at day0, day1, and day3, compared to the number of donors in the pseudotime-inferred mesendo and defendo stages. (**B**) As for **A**, with the number of cells.

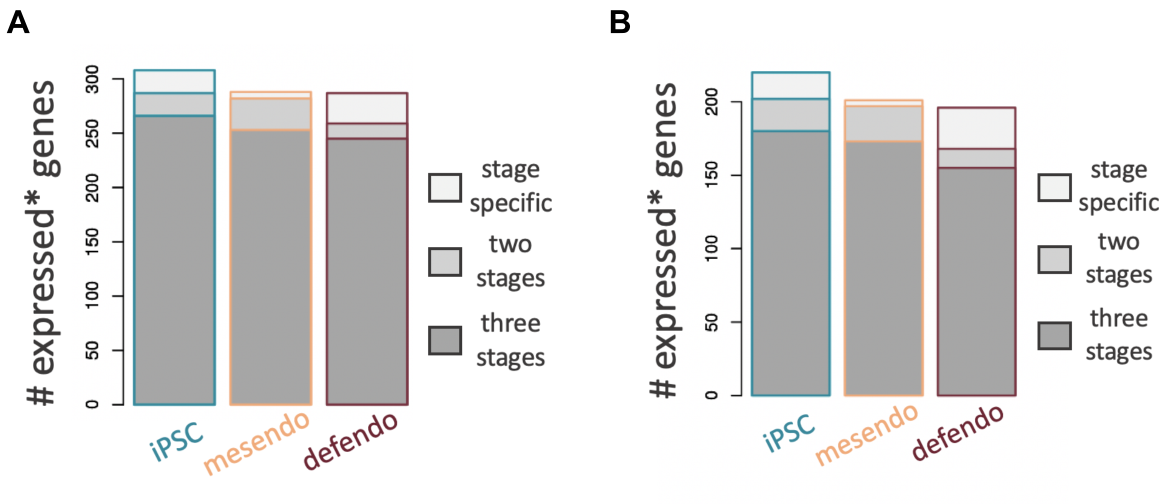

Supplementary Figure 12 | Stage-specific eGenes are mostly expressed across all stages. Similar to Fig. 2C. Proportion of stage-specific eGenes (genes with a stage-specific eQTL) that are expressed (above a threshold T) only at a single stage, expressed at two stages, or expressed at all stages. Expressed is defined as normalised log2(CPM+1) > T. CPM: counts per million. In (**A**) T=1, in (**B**) T=2**.**

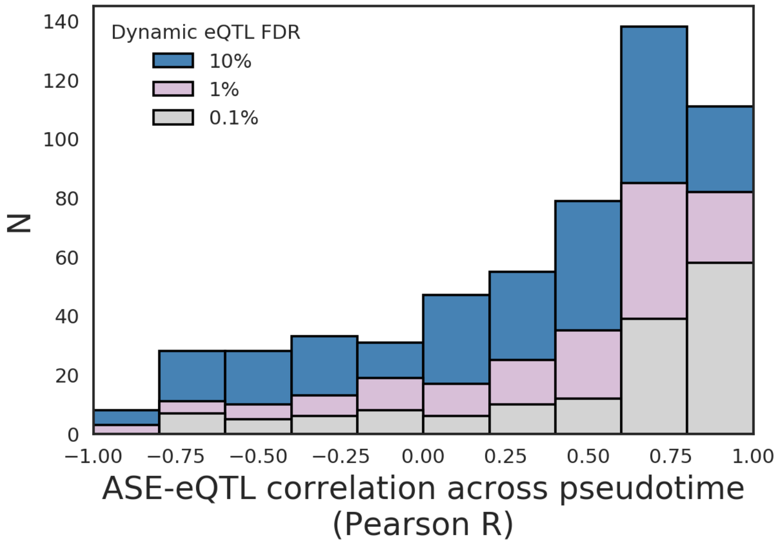

Supplementary Figure 13 | Comparison of eQTL effect and ASE dynamics across pseudotime. The correlation between eQTL effect (i.e. -log10(p) x direction of effect) and ASE across pseudotime, at different FDR thresholds, with 1% FDR corresponding to the set of eQTLs plotted in **Fig 3A**.

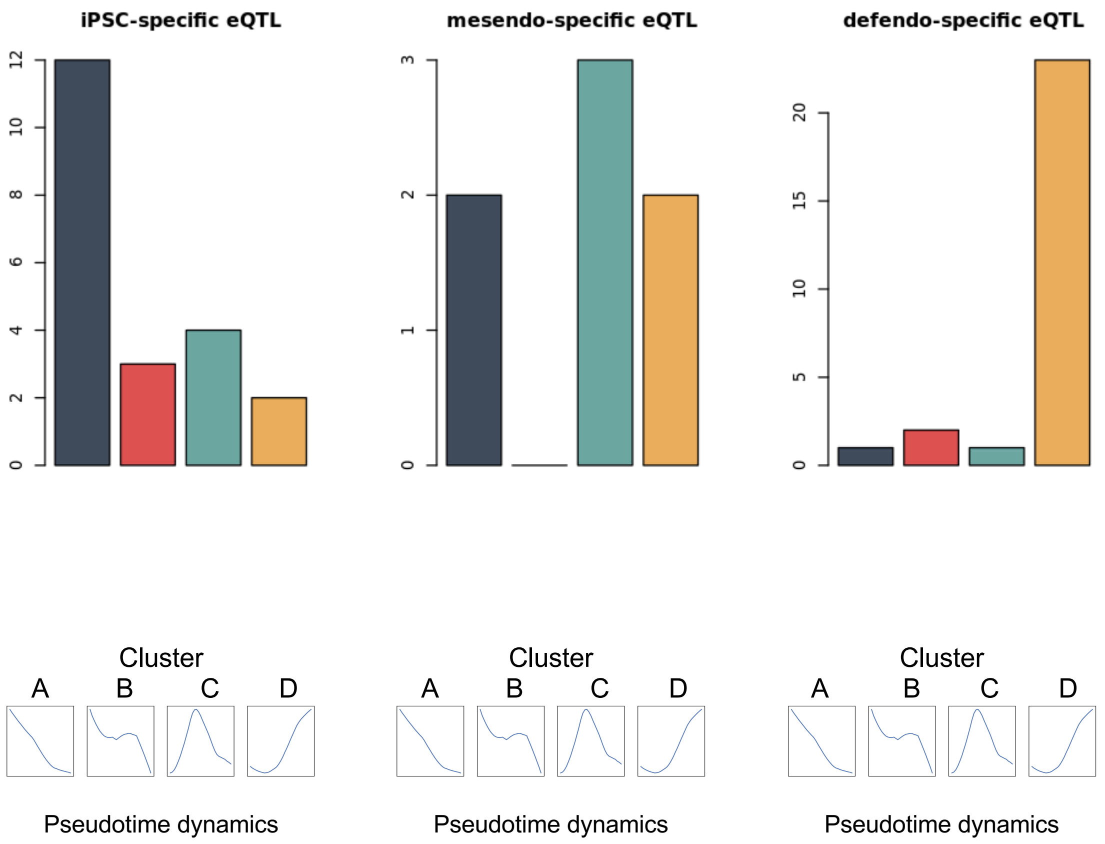

Supplementary Figure 14 | Assignment of stage-specific eQTLs to dynamic eQTL clusters. The numbers of each of the 3 classes of stage-specific eQTL (i.e. iPSC-, mesendo-, and defendo-specific eQTLs) that are assigned to each of the 4 dynamic eQTL clusters.

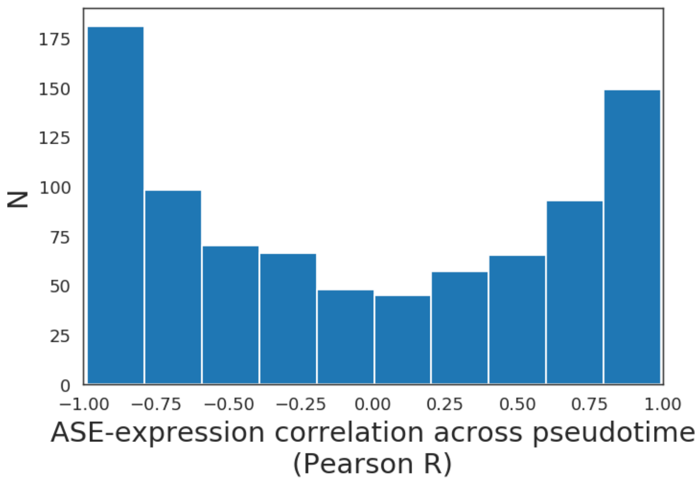

Supplementary Figure 15 | Correlations of ASE with gene expression across pseudotime. ASE and gene expression quantifications were binned across a sliding window of pseudotime as in Fig. 3A. ASE was defined as deviation from balanced allelic expression, such that positive ASE-expression correlations correspond to eQTL for which the eQTL effect is strongest when expression is highest, while negative correlations occur when the eQTL effect is strongest when expression is lowest. Results are shown for the set of 872 dynamic eQTL identified.

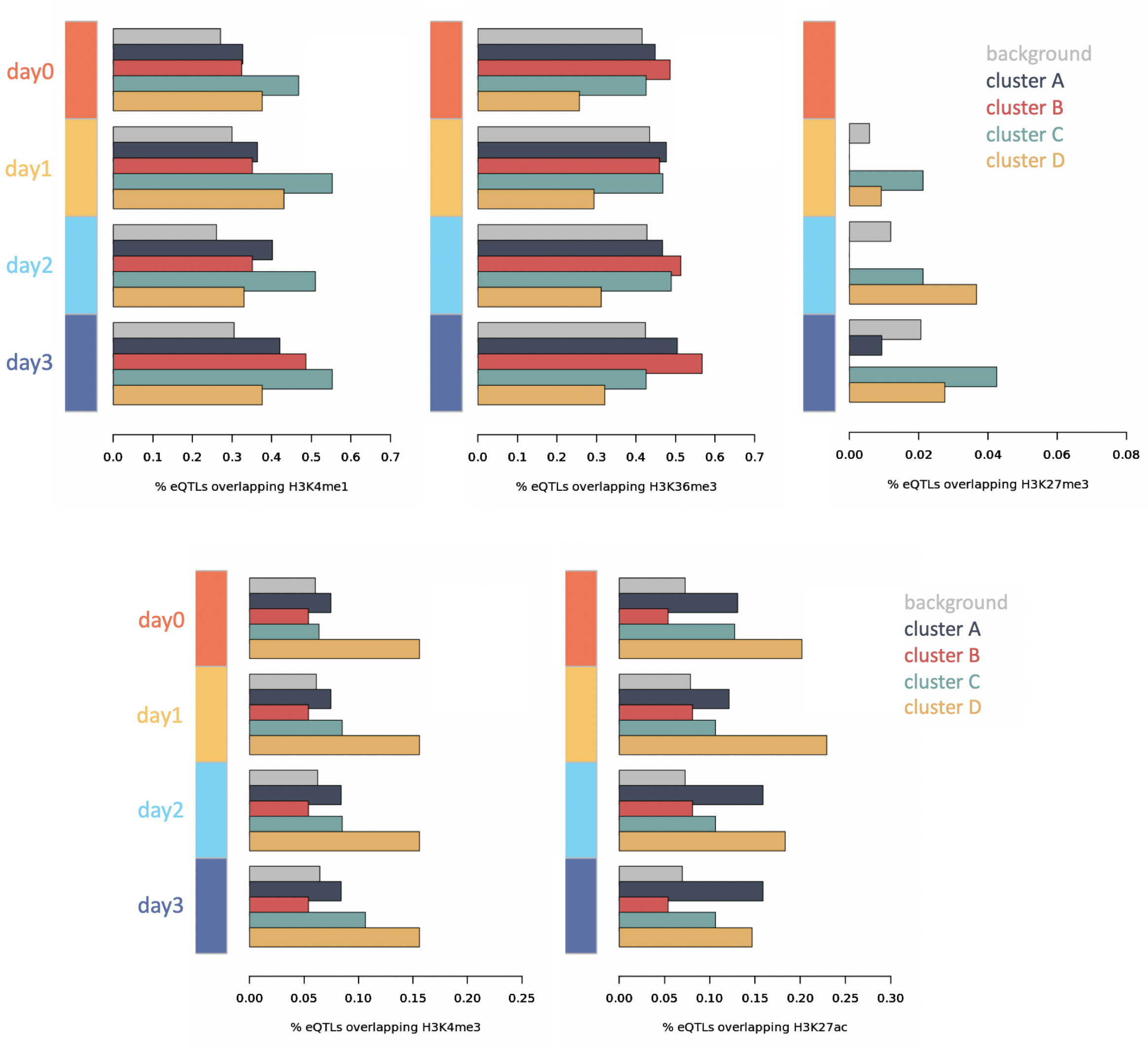

Supplementary Figure 16 | Epigenetic marks of dynamically regulated eQTL SNPs across pseudotime dynamics clusters, and time points. Related to **Fig 3E**. Proportions of dynamic eQTL in each category overlapping each epigenetic mark at each time point are shown. Proportions of overlap with 'background' eQTL (i.e. those without an interaction with pseudotime at FDR 10%) are shown in grey for comparison.

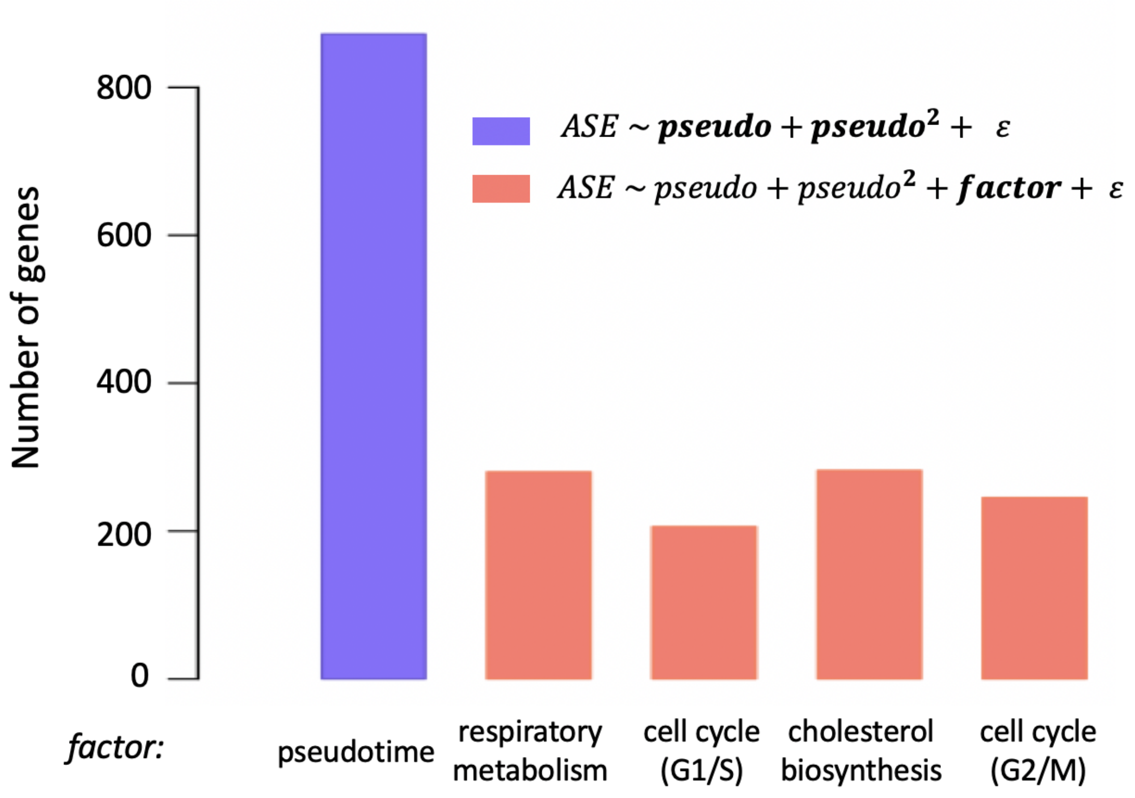

Supplementary Figure 17 | Summary of allele-specific expression interaction test results for each tested cellular state. Results from **Supplementary Data 8, 9, 10.** The number of significant interactions in each category are provided. Bars represent the number of genes with at least one eQTL that is significant for each test described in the inset (**Methods**), FDR < 10%.

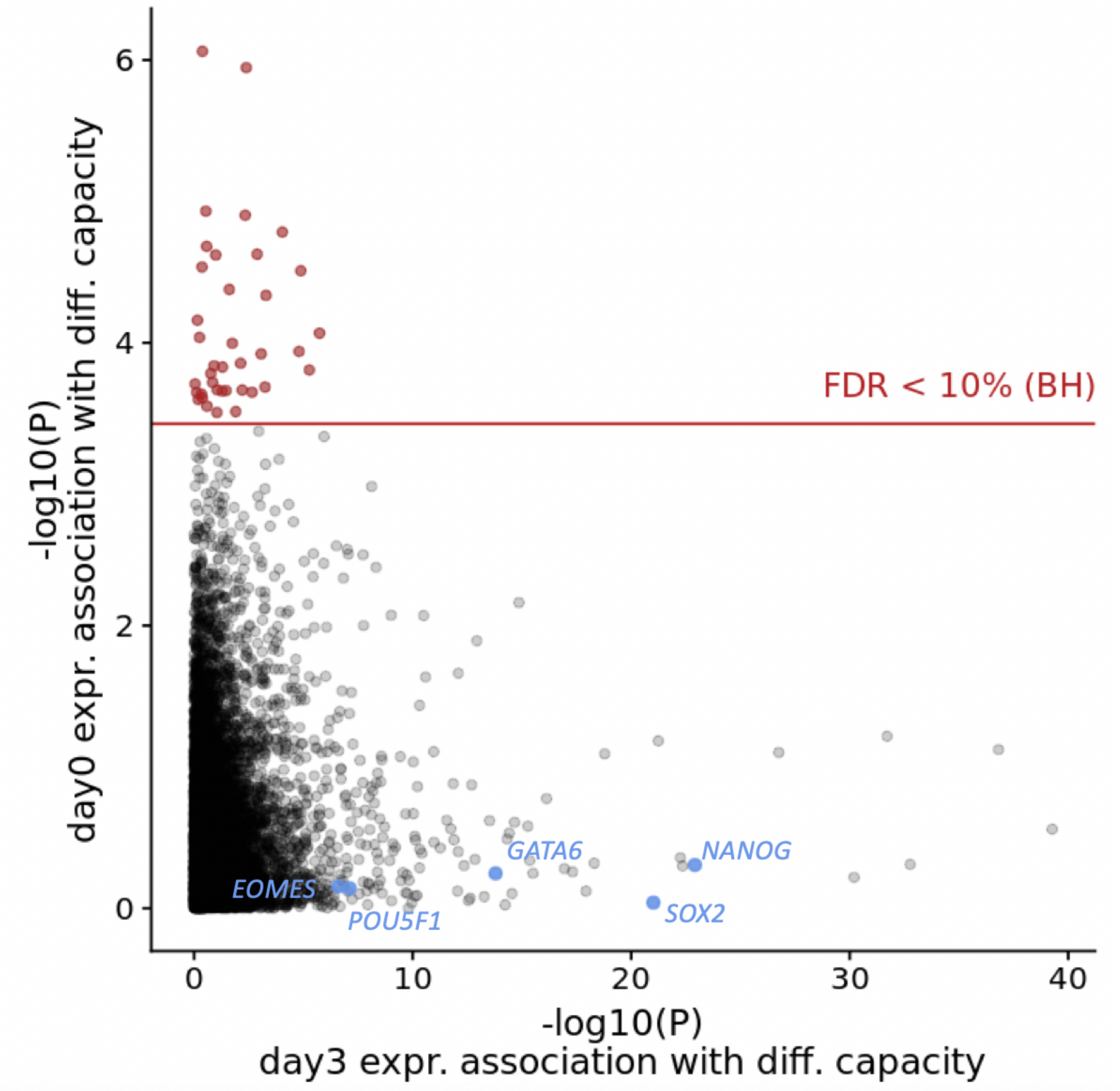

Supplementary Figure 18 | Independence of predictive markers of differentiation capacity from genes that define differentiation capacity (i.e. pseudotime on day3). Scatterplot comparing the statistical significance of associations for all genes. The y-axis shows the association between the expression of the gene on day0 (i.e. in iPSC) and differentiation capacity of cell lines. The x-axis shows the association between the expression of the gene on day3 and differentiation capacity. The correlation between these associations is $\rho = 0.055$, demonstrating that the genes identified as predictive markers of differentiation capacity (i.e. those with high values on the y-axis) are not the genes that define differentiation capacity (i.e. those with high values on the x-axis). Significance measured as -log10(P-value). In red are the 38 genes whose day0 expression is significantly associated with differentiation capacity (FDR < 10%, same genes as in **Fig. 5B**). In blue are a selection of marker genes known to distinguish between pluripotent and definitive endoderm stages of development.

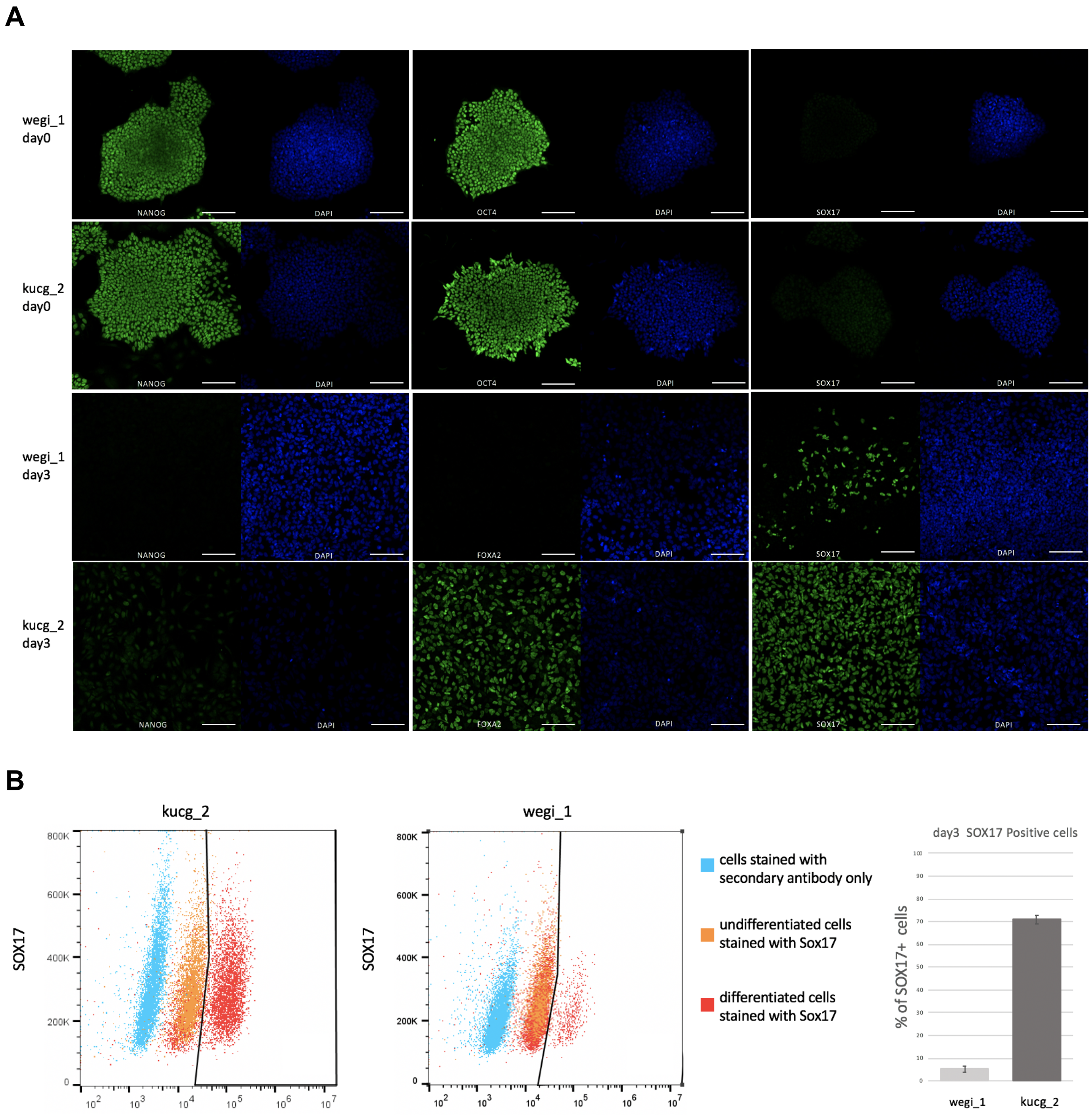
A
wegi_1 day0
kucg_2 day0
wegi_1 day3
kucg_2 day3
NANOG
DAPI
OCT4
SOX17
FOXA2
B
kucg_2
wegi_1
SOX17
800K
600K
400K
200K
0
cells stained with secondary antibody only
undifferentiated cells stained with Sox17
differentiated cells stained with Sox17
day3 SOX17 Positive cells
% of SOX17+ cells
wegi_1
kucg_2

C

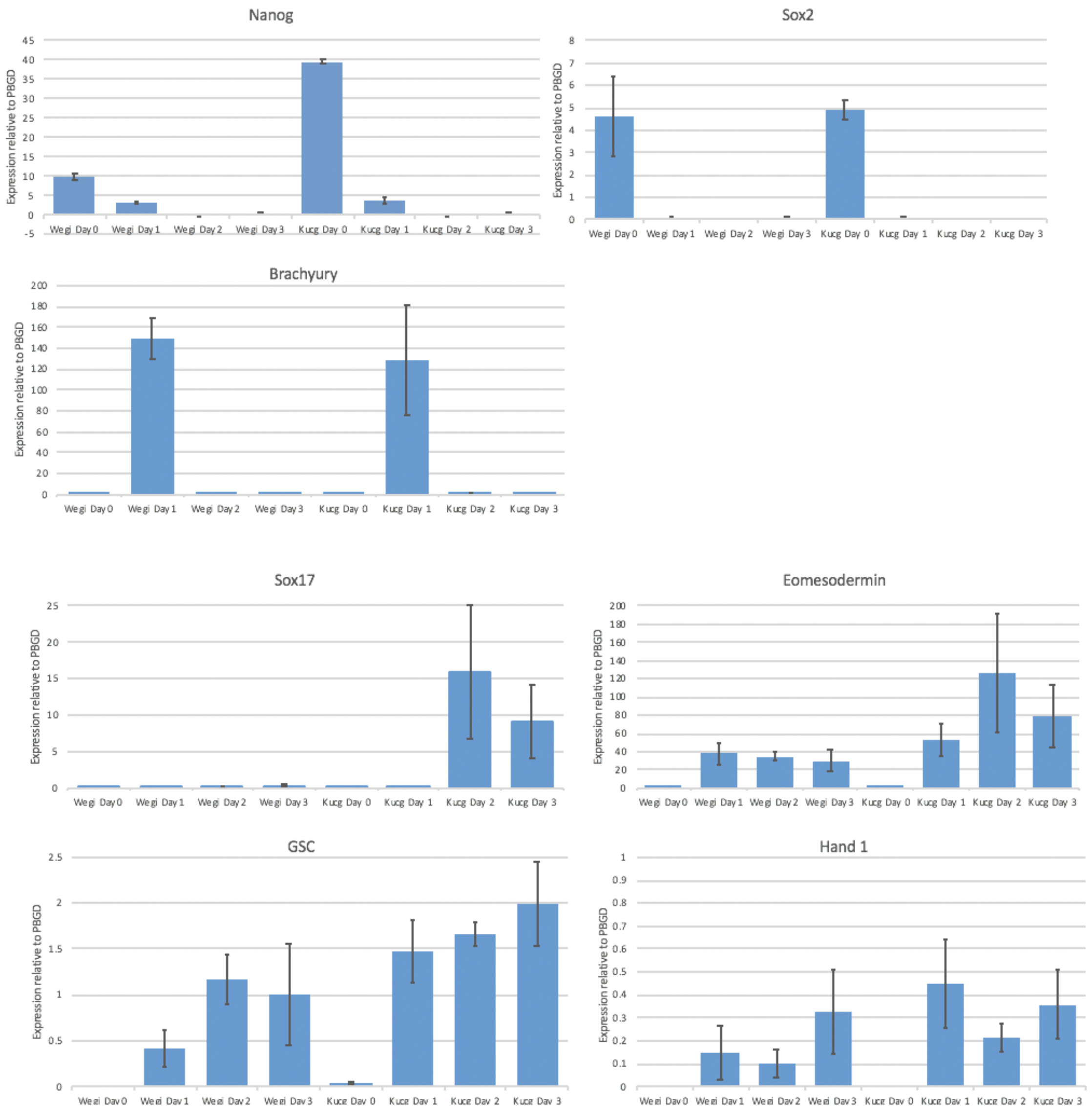

Supplementary Figure 19 | Validation of the definitive endoderm differentiation protocol. wegi_1 and kucg_2 were identified as poor and highly efficient lines, respectively, for definitive endoderm differentiation. Shown is the expression of various markers in the iPSC and differentiated state as assessed by immunofluorescence (**A),** FACS (**B**), and qPCR (**C).**

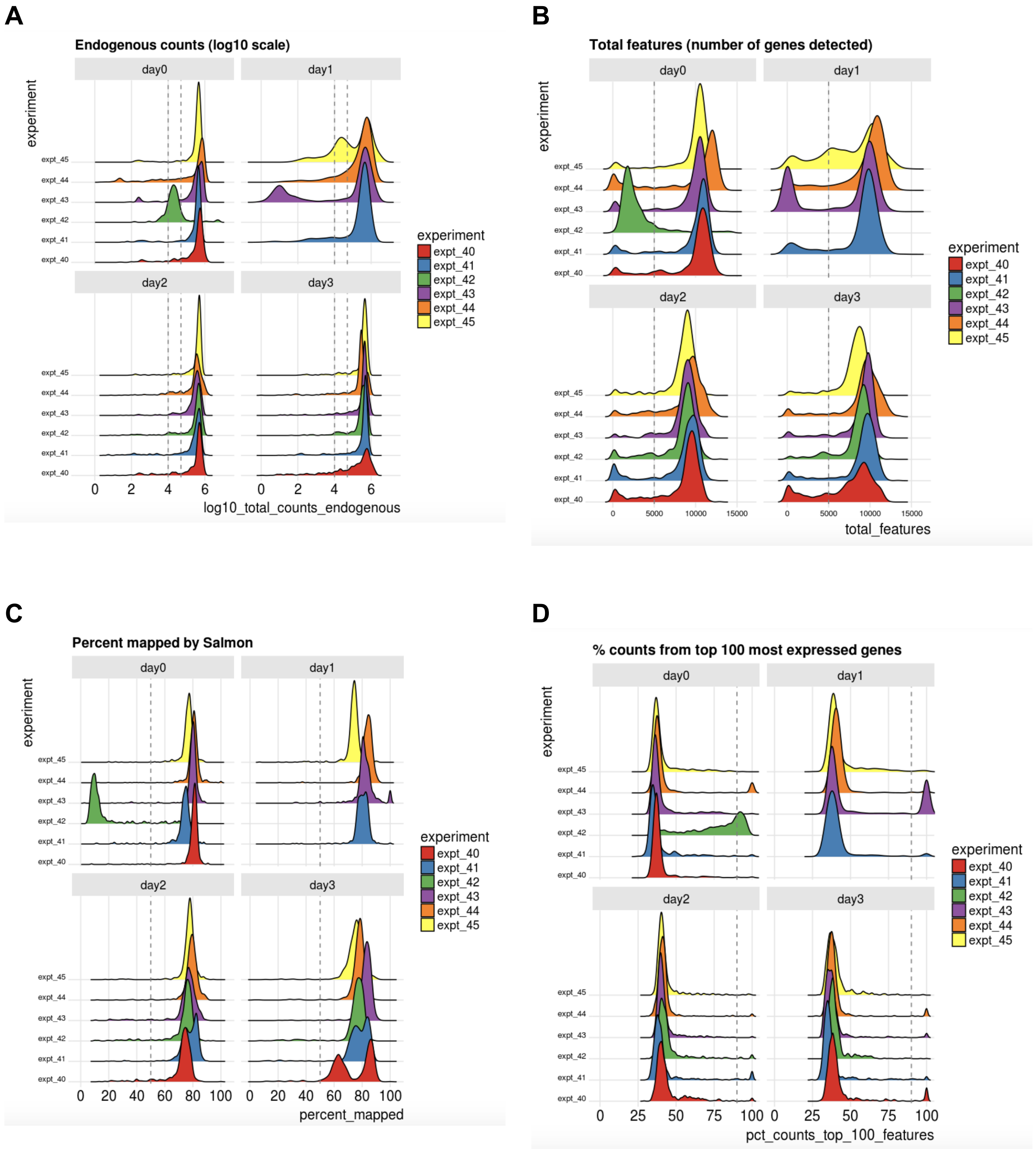
A
Endogenous counts (log10 scale)
day0
day1
day2
day3
experiment
expt_45
expt_44
expt_43
expt_42
expt_41
expt_40
0 2 4 6
log10_total_counts_endogenous
experiment
expt_40
expt_41
expt_42
expt_43
expt_44
expt_45
B
Total features (number of genes detected)
day0
day1
day2
day3
0 5000 10000 15000
total_features
C
Percent mapped by Salmon
0 20 40 60 80 100
percent_mapped
D
% counts from top 100 most expressed genes
0 25 50 75 100
pct_counts_top_100_features

E

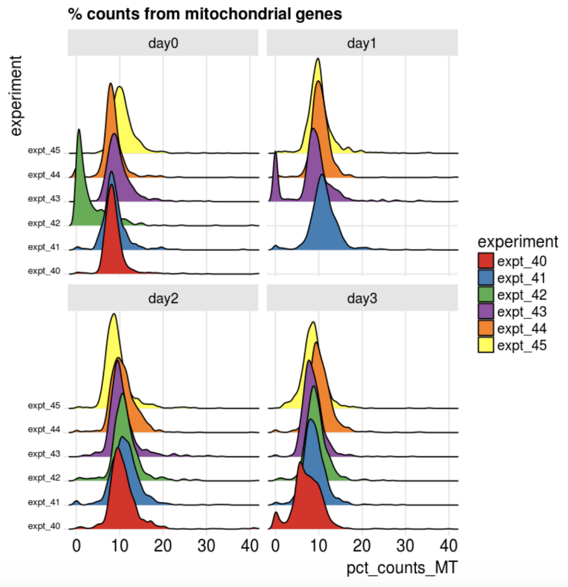

Supplementary Figure 20 | Distributions of quality control metrics across all days in an illustrative subset of 6 differentiation experiments. (**A**) Number of counts for endogenous genes per cell. (**B**) Total number of features (i.e. genes) detected per cell. (**C**) Salmon mapping rate i.e. the percentage of reads successfully mapped to the transcriptome by Salmon. (**D**) Percentages of counts coming from the top 100 most highly expressed genes for each cell. (**E**) Percentage of counts from mitochondrial genes for each cell. In all plots, vertical dashed lines indicate the threshold applied to define the low-quality cells that are excluded from further analysis (**Methods**).

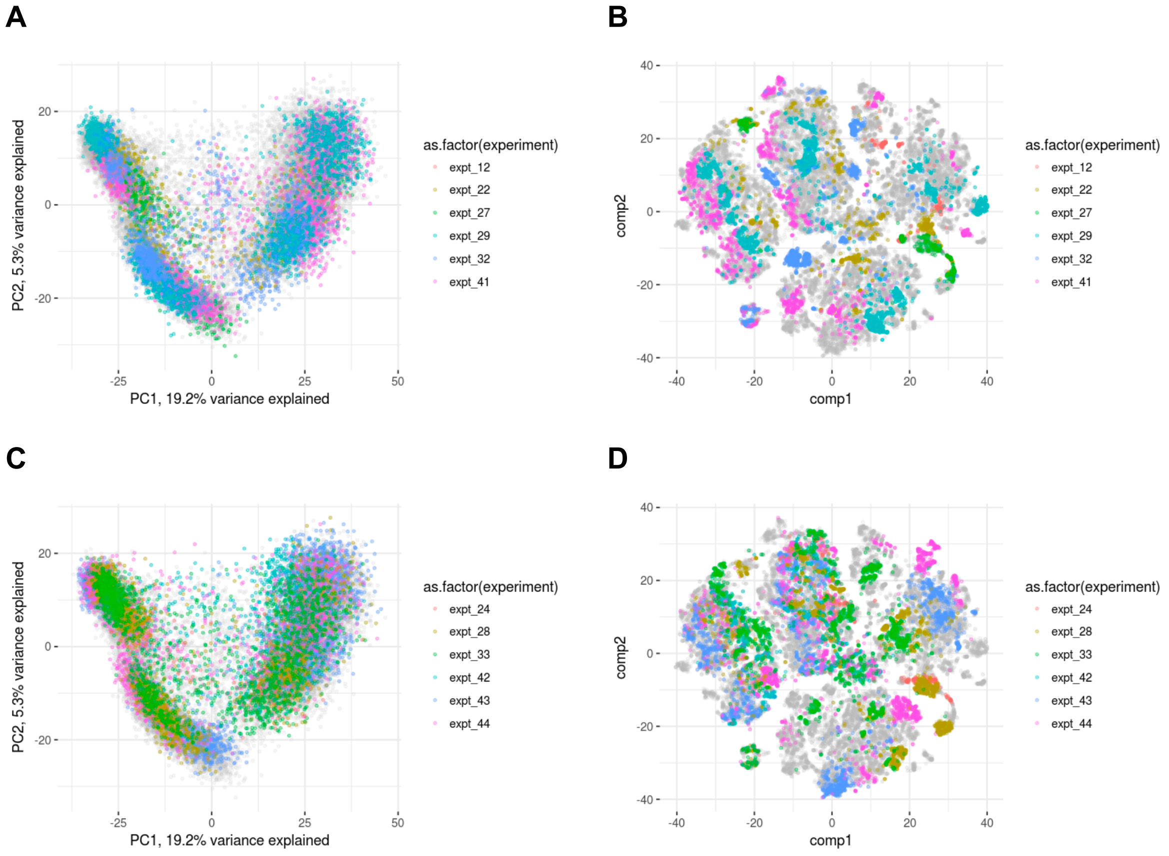

Supplementary Figure 21 | Comparisons of gene expression across experiments. PCA and t-SNE representations of two randomly selected sets of 6 experiments for which data were available across all days. (**A**) PCA plot for the first subset of 6 experiments (colours), against the background of all cells (grey). (**B**) t-SNE plot of the same cells as in **A**. (**C, D**) As for **A, B**, for a different subset of cell differentiation experiments.

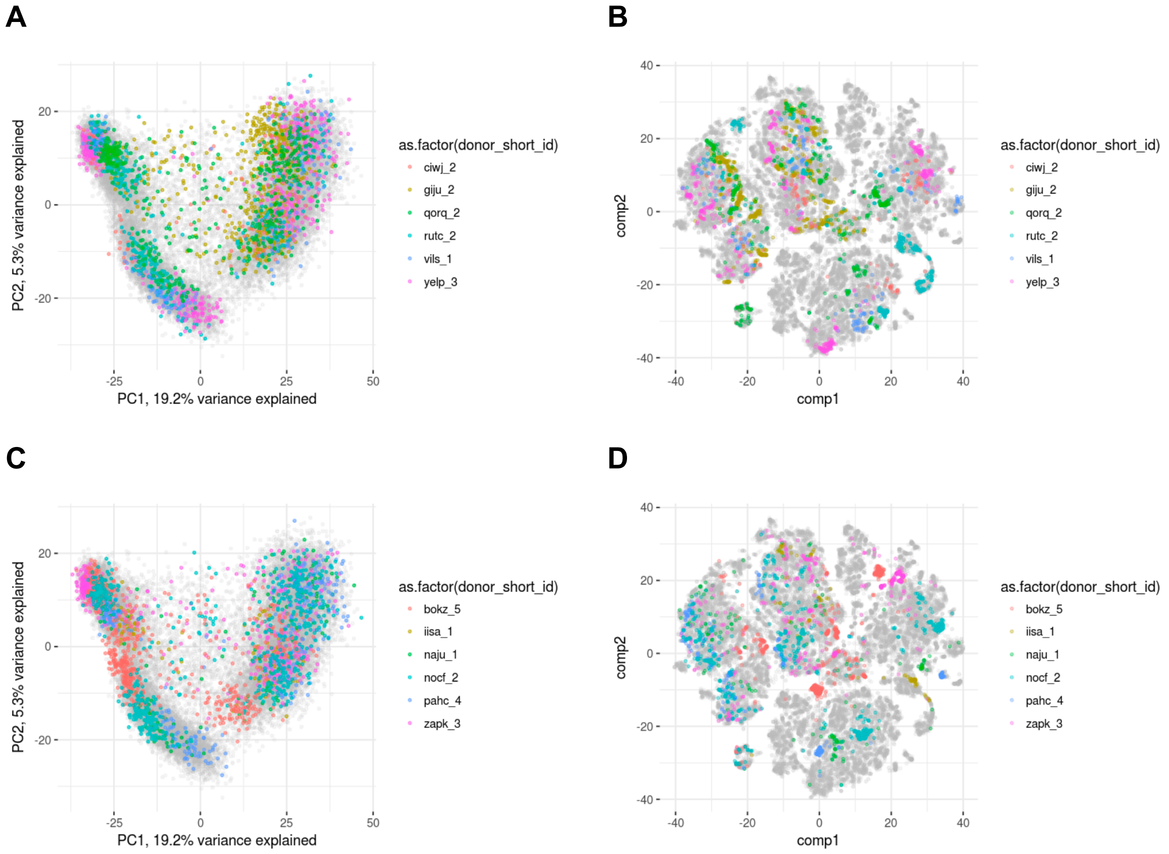

Supplementary Figure 22 | Comparison of expression patterns across cell lines. PCA and t-SNE representations of cells from two randomly selected sets of 6 cell lines. (**A**) PCA plot for the first subset of 6 cell lines (colours), against the background of all cells (grey). (**B**) t-SNE plot of the same cells as in **A**. (**C, D**) As for **A, B**, for a different subset of cell lines.

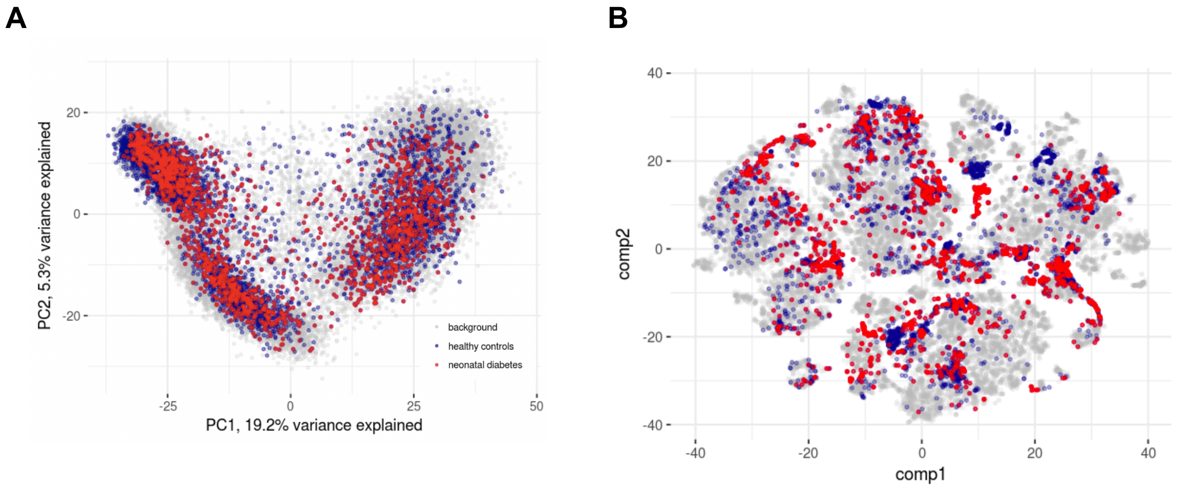

Supplementary Figure 23 | Comparison of expression patterns between healthy and diseased cell lines. PCA and t-SNE representations of cells from neonatal diabetes lines, compared to healthy lines from the same experiments. (**A**) PCA plot for cells from the neonatal diabetes cell lines (red), cells from healthy lines from the same seven experiments (dark blue), against the background of all cells (grey). (**B**) t-SNE plot of the same cells as in **A**.

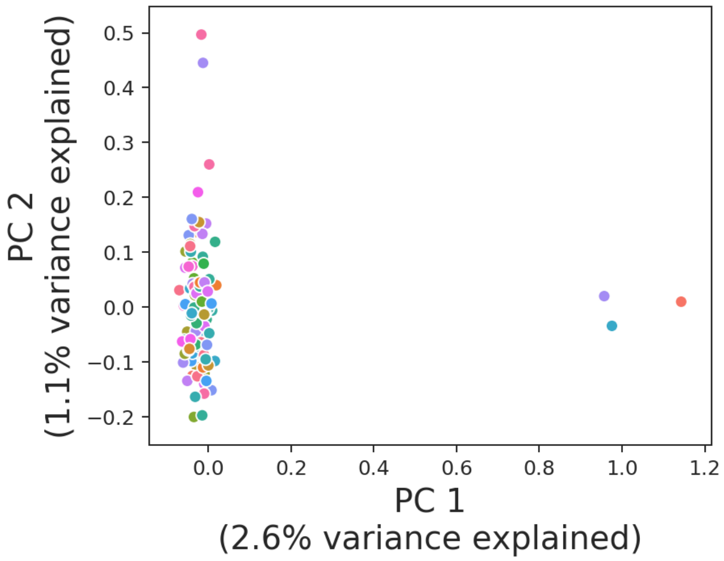

Supplementary Figure 24 | Population structure of donors across experiments. Principal component (PC) decomposition of the kinship matrix across all unique donors, coloured by the experiment in which that donor was included (donors that were included in more than one experiment were assigned the colour of one of those experiments at random).

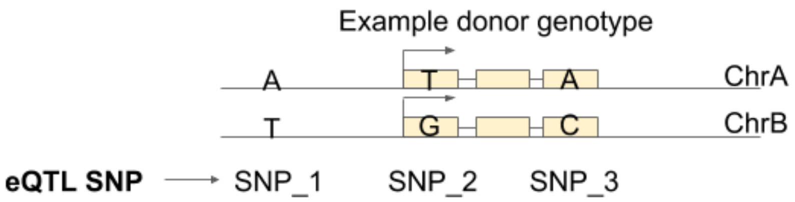

SNP information

SNP	REF	ALT
SNP_1	T	A
SNP_2	G	T
SNP_3	A	C

Genotype information

Donor	SNP_1	SNP_2	SNP_3
Example	1|0	1|0	0|1

1) Count allele-specific reads from RNA-seq

SNP	REF	ALT
SNP_2	11	19
SNP_3	24	16

2) Convert to ChrA/ChrB read counts

SNP	ChrA	ChrB
SNP_2	19	11
SNP_3	24	16

3) Sum counts to gene level

Gene	ChrA	ChrB
Example gene	43	27

4) Define chromosomes relative to alleles of the eQTL SNP

Gene	Chr with the REF eQTL allele	Chr with the ALT eQTL allele
Example gene	27	43

5) Convert to allelic fraction

Gene	Fraction of reads from Chr with the ALT eQTL allele
Example gene	0.61

Supplementary Figure 25 | Worked example of the ASE quantification procedure. A toy example is shown, to illustrate the steps involved in quantifying ASE for an eQTL. ASE is first quantified for SNPs, then combined at gene level, then re-defined relative to the genotype and phase of the eQTL variant. **SNP information:** the REF and ALT alleles. **Genotype information:** the genotype of each individual, including phasing information, in “chrA|chrB” format, where 0 is REF and 1 is ALT (e.g. “0|1” indicates chrA is the REF allele and chrB is the ALT allele)**.**

Supplementary Tables

Supplementary Table 1. Summary of the type and number of eQTL. Including all eQTL discovered based on single cell (at iPS, mesendo, defendo stage, and day0, day1, day3 time point) and bulk (only iPS) RNA traits. Shown are the number of genes that were considered for QTL mapping, as well as the number of genes for which a QTL was detected.

	Number of genes with an eQTL (FDR < 0.1)	Number of genes tested	Number of cells in pool	Sample size (number of donors)	Number of (donor, day, experiment) combinations
bulk iPS	2,908	10,736	-	108	-
sc iPS (day0)	1,833	10,840	9,661	111	136
sc mesendo	1,702	10,924	9,809	123	224
sc defendo	1,342	10,901	10,187	116	238
sc transitioning	227	10,924	6,387	118	313
sc mesendo + some transit	468	10,924	10,420	124	233
sc defendo + some transit	1,217	10,924	15,109	117	242
sc day1	1,181	10,787	9,443	111	138
sc day2	718	10,788	8,455	105	116
sc day3	631	10,765	8,485	108	127

Supplementary Table 2. Functional annotation of clusters. See **Supplementary Data 6, 7** for supporting GO and ChIP-seq enrichment data.

**Cluster label**	**Functional annotation**
0	Respiration
10	G1/S transition
28	Sterol biosynthesis
30	G2/M transition

Supplementary Table 3. Composition of Gelatine, MEF media, CDM-PVA, and RPMI/B27.

**Gelatine (500 mL)**			
**Supplier**	**Catalogue #**	**Product**	**Amount**
Sigma	G1890-100G	Gelatine	0.5 g
Sigma	W1503-500ML	Water for Embryo Transfer	500 mL
**MEF Medium (500 mL)**			
**Supplier**	**Catalogue #**	**Product**	**Amount**
Gibco	12634028	Advanced DMEM F12	450 mL
Biosera	S04253S181S	FBS	50 mL
Invitrogen	25030024	L-Glutamine	5 mL
Gibco	15140122	Penicillin-Streptomycin	5 mL
Sigma	M6250-100ML	b-Mercaptoethanol	3.5 µL
**CDM-PVA (500 mL)**			
**Supplier**	**Catalogue #**	**Product**	**Amount**
Gibco	31765068	F-12	250 mL
Gibco	21980065	IMDM	250 mL
Invitrogen	11905031	Conc. Lipids	5 mL
Sigma	M6145-100ML	MTG	20 µL

Roche	10652202001	Transferrin	250 µL
Roche	11376497001	Insulin	350 µL
Invitrogen	15140122	Penicillin-Streptomycin	5 mL
Sigma	P8136	Poly(vinyl alcohol)	0.5 g
**RPMI/B27**			
**Supplier**	**Catalogue #**	**Product**	**Amount**
Gibco	61870-10	RPMI1640 + GlutMax	500 mL
Invitrogen	17504-044	B27	10 mL
Invitrogen	11140-050	MEM-NEAA	5 mL
Invitrogen	15140122	Penicillin-Streptomycin	5 mL

Supplementary Table 4. Antibodies for Immunofluorescence Staining.

Primary Antibodies			
**Host/Target**	**Supplier**	**Catalogue #**	**Dilution**
Goat anti-human Nanog	R&D Systems	AF1997	1:100
Goat anti-human Oct4	Santa Cruz	sc-8628	1:100
Goat anti-human Sox17	R&D Systems	AF1924	1:200
Goat anti-human FoxA2	R&D Systems	AF2400	1:100
**Secondary Antibodies**			
**Fluorophore/Host/Target**	**Supplier**	**Catalogue #**	**Dilution**
Alexa Fluor 568 Donkey Anti-Goat IgG (H+L)	Invitrogen	A11057	1:1000
Alexa Fluor 568 Donkey Anti-Mouse IgG (H+L)	Invitrogen	A10037	1:1000
Alexa Fluor 568 Donkey Anti-Rabbit IgG (H+L)	Invitrogen	A10042	1:1000
Alexa Fluor 488 Donkey anti-Goat IgG (H+L)	Invitrogen	A11055	1:1000

Alexa Fluor 488 Donkey anti-Mouse IgG (H+L)	Invitrogen	A21202	1:1000
Alexa Fluor 488 Donkey anti-Rabbit IgG (H+L)	Invitrogen	A21206	1:1000
Alexa Fluor 647 Donkey anti-Goat IgG (H+L)	Invitrogen	A21447	1:1000
Alexa Fluor 647 Donkey anti-Mouse IgG (H+L)	Invitrogen	A31571	1:1000
Alexa Fluor 647 Donkey anti-Rabbit IgG (H+L)	Invitrogen	A31573	1:1000

Supplementary Table 5. Primers for RT-qPCR.

Gene	Primer sequence (5’ –> 3’)
NANOG	CATGAGTGTGGATCCAGCTTG (Fwd)
NANOG	CCTGAATAAGCAGATCCATGG (Rev)
SOX2	TGGACAGTTACGCGCACAT (Fwd)
SOX2	CGAGTAGGACATGCTGTAGGT (Rev)
BRACHURY	TGCTTCCCTGAGACCCAGTT (Fwd)
BRACHURY	GATCACTTCTTTCCTTTGCATCAAG (Rev)
EOMESODERMIN	ATCATTACGAAACAGGGCAGGC (Fwd)
EOMESODERMIN	CGGGGTTGGTATTTGTGTAAGG (Rev)
SOX17	CGCACGGAATTTGAACAGTA (Fwd)
SOX17	GGATCAGGGACCTGTCACAC (Rev)
GSC	GAGGAGAAAGTGGAGGTCTGGTT (Fwd)
GSC	CTCTGATGAGGACCGCTTCTG (Rev)
HAND1	GTGCGTCCTTTAATCCTCTTC (Fwd)
HAND1	GTGAGAGCAAGCGGAAAAG (Rev)
PBGD	GGAGCCATGTCTGGTAACGG (Fwd)
PBGD	CCACGCGAATCACTCTCATCT (Rev)

Supplementary Table 6. Antibodies used for ChIP-seq experiments.

**Antibody raised against**	**Catalogue number**	**Company**
Histone H3	ab1791	Abcam
Histone H3 (tri methyl K4)	ab8580	Abcam
Histone H3 (tri methyl K27)	C15200181 (MAb-181-050)	Diagenode
Histone H3 (mono methyl K4)	ab8895	Abcam
Histone H3 (acetyl K27)	ab4729	Abcam
Histone H3 (tri methyl K36)	ab9050	Abcam

Supplementary References

1. Touboul T, Hannan NRF, Corbineau S, Martinez A, Martinet C, Branchereau S, et al. Generation of functional hepatocytes from human embryonic stem cells under chemically defined conditions that recapitulate liver development. Hepatology. 2010;51: 1754–1765.

2. Haghverdi L, Büttner M, Wolf FA, Buettner F, Theis FJ. Diffusion pseudotime robustly reconstructs lineage branching. Nat Methods. 2016;13: 845–848.
