# Supplementary material for: Single-cell RNA-sequencing of differentiating iPS cells reveals dynamic genetic effects on gene expression
Source: Nat Commun. 2020 Feb 10;11:810. doi: 10.1038/s41467-020-14457-z (PMC7010688; doi:10.1038/s41467-020-14457-z)
Supplement: Supplementary file 2 — Description of Additional Supplementary Files [file 41467_2020_14457_MOESM2_ESM.pdf]

Legends for Supplementary Data Files

Title: Supplementary Data 1. Differentiation experiment metadata.
Description: Supplementary Data 1 is supplied as an external data file.

Title: Supplementary Data 2. Cell line and donor metadata.
Description: Supplementary Data 2 is supplied as an external data file.

Title: Supplementary Data 3. Summary of single cell eQTL results
Description: at all stages. All lead eQTL SNP-gene pairs are provided. Supplementary Data 3 is supplied as an external data file with fields defined below.

**Table field**	**Description**
ensembl_gene_id	Ensembl ID (Ensembl version 75)
snp_id	Lead variant, SNP ID in the format [chromosome]_[position]_[reference]_[alternative allele]
p_value	Nominal P-value
empirical_feature_p_value	Gene-level corrected P-value using using 1,000 permutations
global_corr_p_value	Q-value, globally corrected P-value using Storey procedure
beta	Effect size of the eQTL
beta_se	Standard error of the effect size
gene_name	HGNC symbol
snp_chromosome	Variant chromosome
snp_position	Variant position
ref_allele	Variant reference allele
alt_allele	Variant alternative allele
stage	Stage at which the eQTL was discovered
stage_specific	Whether the eQTL is specific to the stage in which it was discovered (True/False)
interaction_qtl	Whether the eQTL is found to be sensitive to any of the measured cell states, including pseudotime (True/False)
dynamic_qtl	Whether the eQTL is found to be sensitive to pseudotime (True/False)
in_HipSci	Whether the eQTL is tagging an iPSC eQTL from [1]

n_gtex_tissues	How many GTEx tissues is the eQTL tagging (0-49)
GWAS_tagging	Whether the eQTL is tagging a GWAS variant

Title: Supplementary Data 4. Summary of bulk iPS eQTL results.
Description: The list of genes with a significant (FDR < 10%) eQTL. Supplementary Data 4 is supplied as an external data file with
fields defined below.

**Table field**	**Description**
ensembl_gene_id	Ensembl ID (Ensembl version 75)
snp_id	Lead variant, SNP ID in the format [chromosome]_[position]_[reference]_[alternative allele]
p_value	Nominal P-value
empirical_feature_p_value	Gene-level corrected P-value using using 1,000 permutations
global_corr_p_value	Q-value, globally corrected P-value using Storey procedure
beta	Effect size of the eQTL
beta_se	Standard error of the effect size
gene_name	HGNC symbol
snp_chromosome	Variant chromosome
snp_position	Variant position
ref_allele	Variant reference allele
alt_allele	Variant alternative allele

Title: Supplementary Data 5. Coexpression clusters.
Description: List of genes (HGNC symbols) and their corresponding coexpression cluster. S upplementary Data 5 is supplied as external data file.

Title: Supplementary Data 6. Gene ontology (GO) enrichments for all clusters
Description: (Fisher's exact test). Supplementary Data 6 is supplied as an external data file. Column key: 'cluster_label': cluster label, 'GO': GO term ID number, 'NS': GO term category, 'enrichment': whether it is an enrichment (e) or depletion (p), 'name': full name of the GO term, 'ratio_in_study': ratio of proteins in the cluster that are annotated with this GO term, 'ratio_in_pop': ratio of all proteins that are annotated with this GO term, 'p_uncorrected': raw p-value from Fisher's exact test, 'depth': depth of GO term in the GO tree, 'study_count': number of proteins in the cluster annotated with this GO term, 'p_fdr_bh': p-value after correction for multiple testing by Benjamini-Hochberg.

Title: Supplementary Data 7. Enrichments of transcription factor binding in coexpression clus ters.
Description: The ChEA 2016 database [2] was used to identify transcription factor target genes.

Title: Supplementary Data 8. **GxE results by ASE analysis.**
Description: Results from linear test as described in (1) from **Methods,** ASE association tests with p seudotime, both linear and quadratic.

Title: Supplementary Data 9. GxE results by ASE analysis.
Description: For all factors, including pseudotime (linear and quadratic) as covariates (2).

Title: Supplementary Data 10. GxE results by ASE analysis.
Description: Non linear interactions, including an interaction between pseudotime and another facto r (3). Pseudotime, pseudotime squared and the factor tested are included as additive covariates.

Supplementary Data 8, 9, 10 are supplied as external data files with fields defined below.

Table field	Description
ensembl_gene_id	Ensembl ID (Ensembl version 75)
snp_id	Lead variant, SNP ID in the format [chromosome]_[position]_[reference]_[alternative allele]
pval	Nominal P-value
ncells	Number of cells considered for ASE
qval	Q-value (following multiple testing correction)
factor	Factor tested (those can be pseudotime, G2_M_transition, sterol_biosynthesis, respiration, G1_S_transition)

Title: Supplementary Data 11. GWAS tagging results.

Description: For the joint set of eQTL identified at iPSC, mesendo, defendo (r^2> 0.8). Suppl ementary Data 11 is supplied as external data file.

Title: Supplementary Data 12. Associations between eQTL variants and differenti ation progress.
Description: Results of association tests between identified eQTL (iPSC, mesendo,defendo) with differentiation progress. Coefficients and p-values of the tests are provided. Supplementary Data 12 is supplied as an external data file.

Title: Supplementary Data 13. Associations between discovered marker genes and differentiation progress.
Description: Related to **Fig. 5B**. Results of association tests between the 38 significantly as sociated genes (FDR < 10%) ("candidate_marker_gene") and differentiation progress. Coeffi cients and nominal p-values for all tests are provided. The column heading suffix ("_all_lines", "_female_lines", "_male_lines") indicates the set of cell lines in which the association test was performed. The chromosome on which each gene is located is also provided. Supplementary Data 13 is supplied as an external data file.

Title: Supplementary Data 14. Variance component results.
Description: Related to **Fig. 1B**. The variance components of cell line, experiment, and time point are provided.

References (Supplementary Data Legends)

1. Mirauta BA, Seaton DD, Bensaddek D, Brenes A, Bonder MJ, Kilpinen H, et al. Population-scale proteome variation in human induced pluripotent stem cells. doi:10.1101/439216

2. Lachmann A, Xu H, Krishnan J, Berger SI, Mazloom AR, Ma'ayan A. ChEA: transcription factor regulation inferred from integrating genome-wide ChIP-X experiments. Bioinformatics. 2010;26: 2438–2444.
