# Supplementary material for: Single-cell RNA-sequencing of differentiating iPS cells reveals dynamic genetic effects on gene expression
Source: Nat Commun. 2020 Feb 10;11:810. doi: 10.1038/s41467-020-14457-z (PMC7010688; doi:10.1038/s41467-020-14457-z)
Supplement: Supplementary file 17 — Reporting Summary [file 41467_2020_14457_MOESM17_ESM.pdf]

nature research

Corresponding author(s): Oliver Stegle, Mariya Chhatriwala, John Marioni, Ludovic Vallier

Last updated by author(s): Dec 5, 2019

Reporting Summary

Nature Research wishes to improve the reproducibility of the work that we publish. This form provides structure for consistency and transparency in reporting. For further information on Nature Research policies, see Authors & Referees and the Editorial Policy Checklist .

Statistics

For all statistical analyses, confirm that the following items are present in the figure legend, table legend, main text, or Methods section.

n/a	Confirmed	
☐	☒	The exact sample size (*n*) for each experimental group/condition, given as a discrete number and unit of measurement
☐	☒	A statement on whether measurements were taken from distinct samples or whether the same sample was measured repeatedly
☐	☒	The statistical test(s) used AND whether they are one- or two-sided<br>*Only common tests should be described solely by name; describe more complex techniques in the Methods section.*
☐	☒	A description of all covariates tested
☐	☒	A description of any assumptions or corrections, such as tests of normality and adjustment for multiple comparisons
☐	☒	A full description of the statistical parameters including central tendency (e.g. means) or other basic estimates (e.g. regression coefficient) AND variation (e.g. standard deviation) or associated estimates of uncertainty (e.g. confidence intervals)
☐	☒	For null hypothesis testing, the test statistic (e.g. *F*, *t*, *r*) with confidence intervals, effect sizes, degrees of freedom and *P* value noted<br>*Give P values as exact values whenever suitable.*
☒	☐	For Bayesian analysis, information on the choice of priors and Markov chain Monte Carlo settings
☒	☐	For hierarchical and complex designs, identification of the appropriate level for tests and full reporting of outcomes
☐	☒	Estimates of effect sizes (e.g. Cohen's *d*, Pearson's *r*), indicating how they were calculated

Our web collection on statistics for biologists contains articles on many of the points above.

Software and code

Policy information about availability of computer code

Data collection	*Provide a description of all commercial, open source and custom code used to collect the data in this study, specifying the version used OR state that no software was used.*
Data analysis	Published software: a combination of NGS analysis tools (Salmon, STAR, GATK, BWA), R (bioconductor) and Python (see details in Methods)<br>Unpublished data analysis scripts will be made available at https://github.com/annacuomo/singlecell_endodiff_paper

For manuscripts utilizing custom algorithms or software that are central to the research but not yet described in published literature, software must be made available to editors/reviewers. We strongly encourage code deposition in a community repository (e.g. GitHub). See the Nature Research guidelines for submitting code & software for further information.

Data

Policy information about availability of data

All manuscripts must include a data availability statement. This statement should provide the following information, where applicable:

- Accession codes, unique identifiers, or web links for publicly available datasets
- A list of figures that have associated raw data
- A description of any restrictions on data availability

We have provided accession codes for sequencing data for all open-access samples in our study. A subset of our human iPSC lines are under controlled access, and will be made available through the EGA. These sequencing data underlie all figures (1-5) in the manuscript.

Field-specific reporting

Please select the one below that is the best fit for your research. If you are not sure, read the appropriate sections before making your selection.

[x] Life sciences [ ] Behavioural & social sciences [ ] Ecological, evolutionary & environmental sciences

For a reference copy of the document with all sections, see nature.com/documents/nr-reporting-summary-flat.pdf

Life sciences study design

All studies must disclose on these points even when the disclosure is negative.

Sample size	Sample size was based on results from many previous human eQTL studies, where above ~80 samples provides power to map eQTL, with power increasing with sample size.
Data exclusions	Single-cell RNA-seq data were excluded based on quality control criteria, as described in the Methods.
Replication	The differentiation assay was repeated across 28 experiment, and its robustness validated by RNA- and protein-based approaches. We also replicate eQTL results across two different single cell RNA-sequencing technologies
Randomization	iPSC lines were allocated to groups/experimental batches at random.
Blinding	Investigators were blinded during collection, as the origin of each assayed cell was only determined during data analysis (by assessment of RNA-sequencing data).

Reporting for specific materials, systems and methods

We require information from authors about some types of materials, experimental systems and methods used in many studies. Here, indicate whether each material, system or method listed is relevant to your study. If you are not sure if a list item applies to your research, read the appropriate section before selecting a response.

Materials & experimental systems

n/a	Involved in the study	
[ ]	[x]	Antibodies
[ ]	[x]	Eukaryotic cell lines
[x]	[ ]	Palaeontology
[x]	[ ]	Animals and other organisms
[x]	[ ]	Human research participants
[x]	[ ]	Clinical data

Methods

n/a	Involved in the study	
[ ]	[x]	ChIP-seq
[ ]	[x]	Flow cytometry
[x]	[ ]	MRI-based neuroimaging

Antibodies

Antibodies used	See Supplementary Table S15
Validation	*Describe the validation of each primary antibody for the species and application, noting any validation statements on the manufacturer's website, relevant citations, antibody profiles in online databases, or data provided in the manuscript.*

Eukaryotic cell lines

Policy information about cell lines

Cell line source(s)	All cell lines are iPSCs from the HipSci project (www.hipsci.org)
Authentication	All cell lines were matched to the original donor by genotype, using RNA-sequencing data.
Mycoplasma contamination	Confirmed
Commonly misidentified lines (See ICLAC register)	NA

ChIP-seq

Data deposition

☒ Confirm that both raw and final processed data have been deposited in a public database such as GEO.

☒ Confirm that you have deposited or provided access to graph files (e.g. BED files) for the called peaks.

Data access links<br>*May remain private before publication.*	PRJNA593217
Files in database submission	See GEO for a complete list (many files)
Genome browser session<br>(e.g. UCSC)	NA

Methodology

Replicates	Two technical replicates were generated per time point, per histone modification.
Sequencing depth	37bn reads were generated in total, sequenced using an Illumina HiSeq 2000 with 75bp paired-end reads.
Antibodies	See Supplementary Table S20
Peak calling parameters	tool peakranger 1.18 in modes ranger (H3K4me3, H3K27ac; '-l 316 -b 200 -q 0.05'), ccat (H3K27me3; '-l 316 --win_size 1000 --win_step 100 --min_count 70 --min_score 7 -q 0.05') and bcp (H3K4me1, H3K36me3; '-l 316'). Adjacent peak regions closer than 40 bp were merged using the BEDTools suite
Data quality	Only reads with mapping quality score ≥ 10 and aligned to autosomal and sex chromosomes were kept for further processing.
Software	BWA, peakranger, BEDTools

Flow Cytometry

Plots

Confirm that:

☒ The axis labels state the marker and fluorochrome used (e.g. CD4-FITC).

☒ The axis scales are clearly visible. Include numbers along axes only for bottom left plot of group (a 'group' is an analysis of identical markers).

☒ All plots are contour plots with outliers or pseudocolor plots.

☒ A numerical value for number of cells or percentage (with statistics) is provided.

Methodology

Sample preparation	Cells were washed twice in D-PBS and incubated in Accutase for 5 min at 37°C. The Accutase was neutralised by adding double its volume of 5% FBS diluted in D-PBS and the cells were fully dissociated by gentle pipetting. Cells were washed twice in D-PBS then fixed by re-suspending in 500 µl of 4% PFA solution diluted in D-PBS for 20 min on ice. Fixed cells were washed twice in D-PBS + 2% FBS and then stored at 4°C in BD stain buffer (BD Pharmingen Cat # 554656) for up to a week prior to analysis.
Instrument	BD LRSFortessa cell analyser (BD Biosciences)
Software	FlowJo
Cell population abundance	*Describe the abundance of the relevant cell populations within post-sort fractions, providing details on the purity of the samples and how it was determined.*
Gating strategy	*Describe the gating strategy used for all relevant experiments, specifying the preliminary FSC/SSC gates of the starting cell population, indicating where boundaries between "positive" and "negative" staining cell populations are defined.*

☒ Tick this box to confirm that a figure exemplifying the gating strategy is provided in the Supplementary Information.
